# Supplementary material for: Kaempferol-3-O-Glucuronide Ameliorates Non-Alcoholic Steatohepatitis in High-Cholesterol-Diet-Induced Larval Zebrafish and HepG2 Cell Models via Regulating Oxidation Stress
Source: Life (Basel). 2021 May 14;11(5):445. doi: 10.3390/life11050445 (PMC8155963; doi:10.3390/life11050445)
Supplement: Supplementary file 1 [file life-11-00445-s001.zip › supplementary file.pdf]

life

Supplementary Materials:

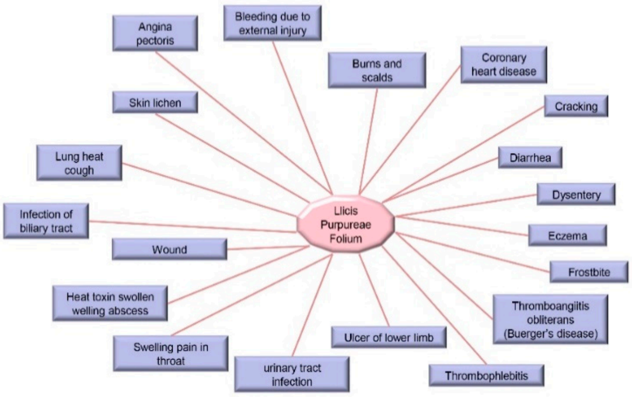

Figure S1. Ilicis Purpureae Folium related disease from network pharmacology. Based on the network pharmacology, the herb and disease relationship is shown in this picture.

Table S1. Ilicis Purpureae Folium related compounds and compound-target relation from network pharmacology.

Herb	Compounds
Ilicis Purpureae Folium	Ilexoside B Methyl Ester
Ilicis Purpureae Folium	3β-O-(β-D-xylopyranosyl)pomolic acid methyl ester
Ilicis Purpureae Folium	cyclohexanone pedunculosyl-3
Ilicis Purpureae Folium	23-o-acetal
Ilicis Purpureae Folium	2'-(o,m-dihydroxybenzyl)sweroside
Ilicis Purpureae Folium	rotundifoline
Ilicis Purpureae Folium	myristic acid
Ilicis Purpureae Folium	Pegaline
Ilicis Purpureae Folium	(2R,4S)-4-hydroxypipecolinic acid
Ilicis Purpureae Folium	odontoside
Ilicis Purpureae Folium	Protocatechuic acid-3-glucoside
Ilicis Purpureae Folium	Rotundic acid
Ilicis Purpureae Folium	siaresinolic acid
Ilicis Purpureae Folium	Isovanillic acid
Ilicis Purpureae Folium	pegamine
Ilicis Purpureae Folium	Ziyuglycoside I
Ilicis Purpureae Folium	ursonic acid
Ilicis Purpureae Folium	[(2S,3R,4S,5S,6R)-3,4,5-trihydroxy-6-(hydroxymethyl)oxan-2-yl] (1R,2R,4aS,6aR,6aS,6bR,8aR,9R,10S,12aR,14bS)-1,10-dihydroxy-9-(hydroxymethyl)-1,2,6a,6b,9,12a-hexamethyl-2,3,4,5,6,6a,7,8,8a,10,11,12,13,14b-tetradecahydropicene-4a-carboxylate
Ilicis Purpureae Folium	Caffeate
Ilicis Purpureae Folium	Geranylacetone
Ilicis Purpureae Folium	Cinnamic acid, 3,4-dimethoxy- (8CI)
Ilicis Purpureae Folium	Tannin
Ilicis Purpureae Folium	Heriguard
Ilicis Purpureae Folium	(6R,10R)-6,10,14-trimethylpentadecan-2-one
Ilicis Purpureae Folium	ilexoside b methyl ester
Ilicis Purpureae Folium	(1S,4aR,6aR,6aS,6bR,8aR,10S,12aR,14bS)-1-hydroxy-2,2,6a,6b,9,9,12a-heptamethyl-10-[(2S,3R,4S,5R)-3,4,5-trihydroxyoxan-2-yl]oxy-1,3,4,5,6,6a,7,8,8a,10,11,12,13,14b-tetradecahydropicene-4a-carboxylic acid
Ilicis Purpureae Folium	β-sitosterol
Ilicis Purpureae Folium	kaempferol-3-O-glucuronide
Ilicis Purpureae Folium	WLN: Q1R
Ilicis Purpureae Folium	syringin
Ilicis Purpureae Folium	GENOP
Ilicis Purpureae Folium	6,6'-dimethoxygossypol
Ilicis Purpureae Folium	caprylic acid
Ilicis Purpureae Folium	Pomolic acid
Ilicis Purpureae Folium	Pomolic acid acetate
Ilicis Purpureae Folium	quercetin
Ilicis Purpureae Folium	apigenin
Ilicis Purpureae Folium	Sinapyl alcohol
Ilicis Purpureae Folium	Stigmasterol
Ilicis Purpureae Folium	kaempferol
Ilicis Purpureae Folium	Trifolin
Ilicis Purpureae Folium	Syrigin
Ilicis Purpureae Folium	Daucosterol_qt
Ilicis Purpureae Folium	PEL
Ilicis Purpureae Folium	3-hexenal
Ilicis Purpureae Folium	(7aR)-4,4,7a-trimethyl-6,7-dihydro-5H-benzofuran-2-one
Ilicis Purpureae Folium	ursolic acid
Ilicis Purpureae Folium	ZINC02038914
Ilicis Purpureae Folium	caffeic acid
Ilicis Purpureae Folium	protocatechuic acid

Ilicis Purpureae Folium	4-Formyl-2,6-xylenol
Ilicis Purpureae Folium	Veratral
Ilicis Purpureae Folium	protocatechualdehyde
Ilicis Purpureae Folium	hexanoic acid
Ilicis Purpureae Folium	palmitic acid
**Compound and target relation**	
**mol_name**	**gene_name**
myristic acid	PPARA
myristic acid	PAEP
myristic acid	PTGS2
myristic acid	PRKACA
myristic acid	PRKCA
myristic acid	PRKCB
myristic acid	PRKCD
myristic acid	PRKCE
myristic acid	PRKCG
myristic acid	PRKCQ
myristic acid	PRKACA
myristic acid	RCVRN
myristic acid	RHO
myristic acid	SEC14L2
myristic acid	SEC14L2
myristic acid	SEC14L2
myristic acid	PPP3CA
myristic acid	PPP3CA
myristic acid	ALB
myristic acid	SLC22A8
myristic acid	SLC22A11
myristic acid	SLC22A6
myristic acid	SLC22A8
myristic acid	APOM
myristic acid	ARF6
myristic acid	TLR4
myristic acid	TRAPPC3
myristic acid	TRAPPC3
myristic acid	GLTP
myristic acid	HNF4A
myristic acid	ABL1
myristic acid	ABL1
myristic acid	ABCB1
myristic acid	ABCB1
myristic acid	ABCB1
myristic acid	XDH
myristic acid	ABL1
myristic acid	ABL1
myristic acid	PPP3R1
myristic acid	PRKACA
myristic acid	PKIA
myristic acid	PKIA
myristic acid	CYP2C8
myristic acid	DCD
myristic acid	ABL1
myristic acid	ECI2
myristic acid	FABP4
myristic acid	FABP3
myristic acid	FABP5
myristic acid	FABP5
myristic acid	FABP4
myristic acid	FABP3

myristic acid	FABP2
myristic acid	FKBP1A
myristic acid	FURIN
myristic acid	FURIN
myristic acid	GM2A
myristic acid	PAEP
myristic acid	GLTP
myristic acid	GLTP
myristic acid	GLTP
myristic acid	PLA2G2D
myristic acid	GUCA1A
myristic acid	HNF4A
myristic acid	HNF4A
myristic acid	HNF4G
myristic acid	HNF4A
myristic acid	HNF4G
myristic acid	INS
myristic acid	ARF1
myristic acid	ARF1
myristic acid	ARF6
myristic acid	LY96
myristic acid	LY96
myristic acid	ALB
myristic acid	ABCB1
myristic acid	PMP2
myristic acid	NCOA1
myristic acid	ODC1
myristic acid	PPT1
myristic acid	FKBP1A
myristic acid	FKBP1A
myristic acid	PPARA
myristic acid	PPARG
myristic acid	ABCB1
myristic acid	LCAT
myristic acid	PLA2G2C
myristic acid	PLA2G1B
myristic acid	PLA2G2A
myristic acid	PVR
myristic acid	GM2A
Isovanillic acid	COMT
Caffeate	TYR
Geranylacetone	ANKRD45
Geranylacetone	HSPA9
Geranylacetone	HSPA1A
Geranylacetone	HSPA5
Geranylacetone	HSPA12A
Geranylacetone	HSPA12B
Geranylacetone	HSPA13
Geranylacetone	HSPA14
Geranylacetone	HSPA1B
Geranylacetone	HSPA1L
Geranylacetone	HSPA4
Geranylacetone	HSPA4L
Geranylacetone	HSPA6
Geranylacetone	HSPA8
Geranylacetone	HSPH1
Geranylacetone	HSPA2
Tannin	ANO1
Tannin	SLCO1B1

Tannin	SLCO1B3
Tannin	ALOX12B
Tannin	ALOX12
Tannin	ALOX15
Tannin	TBXAS1
Tannin	ALOX15B
Tannin	ALOX5
Tannin	SI
Tannin	AKR1E2
Tannin	FYN
Tannin	LCK
Tannin	AKR1D1
Tannin	CLPB
Tannin	CYP1A2
Tannin	ALOXE3
Tannin	GCLM
Tannin	AKR1A1
Tannin	MAPK1
Tannin	MAPK14
Tannin	AKR1B10
Tannin	AKR1B15
Tannin	AKR1C1
Tannin	AKR1C2
Tannin	AKR1C3
Tannin	AKR1C4
Tannin	AKR1B1
β -sitosterol	PRKCA
β -sitosterol	PON1
β -sitosterol	APOE
β -sitosterol	BAX
β -sitosterol	BCL2
β -sitosterol	SREBF1
β -sitosterol	SREBF2
β -sitosterol	JUN
β -sitosterol	TGFB1
β -sitosterol	ABCB11
β -sitosterol	CASP3
β -sitosterol	CASP8
β -sitosterol	CASP9
β -sitosterol	DHCR24
β -sitosterol	ICAM1
β -sitosterol	MAP2
WLN: Q1R	CYP2E1
WLN: Q1R	CES4A
WLN: Q1R	CES5A
WLN: Q1R	N/A
WLN: Q1R	CYP2D6
WLN: Q1R	CYP2D6
WLN: Q1R	CYP2D6
WLN: Q1R	CYP2E1
WLN: Q1R	CYP1A1
WLN: Q1R	CYP3A4
WLN: Q1R	CYP2B6
WLN: Q1R	CYP1A1
WLN: Q1R	CYP1A2
WLN: Q1R	CYP2B6
WLN: Q1R	CYP2C8

WLN: Q1R	CYP2D6
WLN: Q1R	CYP2E1
WLN: Q1R	CYP3A4
WLN: Q1R	CYP3A4
WLN: Q1R	CYP2D6
WLN: Q1R	CYP2B6
WLN: Q1R	CYP2D6
WLN: Q1R	CYP2E1
WLN: Q1R	CYP2D6
WLN: Q1R	CYP2E1
WLN: Q1R	GRIN3A
WLN: Q1R	GRIN3B
WLN: Q1R	GRIN2A
WLN: Q1R	GRIN2C
WLN: Q1R	GRIN2D
WLN: Q1R	CES1
WLN: Q1R	LYZ
WLN: Q1R	ALDH2
WLN: Q1R	ALDH2
WLN: Q1R	ALDH2
WLN: Q1R	ALDH2
WLN: Q1R	CYP2D6
WLN: Q1R	CYP2D6
WLN: Q1R	CYP2D6
GENOP	PTGS1
GENOP	PTGS2
GENOP	SULT1A1
GENOP	CA2
GENOP	FGF1
GENOP	G6PD
GENOP	AOX1
caprylic acid	PPARA
caprylic acid	PAEP
caprylic acid	PRKACA
caprylic acid	PRKACA
caprylic acid	LIPT2
caprylic acid	PDHA1
caprylic acid	RCVRN
caprylic acid	RBP2
caprylic acid	RHO
caprylic acid	SEC14L2
caprylic acid	SEC14L2
caprylic acid	SEC14L2
caprylic acid	PPP3CA
caprylic acid	PPP3CA
caprylic acid	ALB
caprylic acid	SLC22A8
caprylic acid	SLC22A11
caprylic acid	SLC22A6
caprylic acid	SLC22A8
caprylic acid	APOM
caprylic acid	ARF6
caprylic acid	TLR4
caprylic acid	TRAPPC3
caprylic acid	TRAPPC3
caprylic acid	GLTP
caprylic acid	HNF4A
caprylic acid	ABL1
caprylic acid	ABL1

caprylic acid	ABCB1
caprylic acid	ABCB1
caprylic acid	ABCB1
caprylic acid	ABL1
caprylic acid	OXSM
caprylic acid	ABL1
caprylic acid	PPP3R1
caprylic acid	PRKACA
caprylic acid	PKIA
caprylic acid	PKIA
caprylic acid	ALDH9A1
caprylic acid	CYP2C8
caprylic acid	ABL1
caprylic acid	ACACB
caprylic acid	ECI2
caprylic acid	FABP4
caprylic acid	FABP3
caprylic acid	FABP5
caprylic acid	FABP5
caprylic acid	FABP4
caprylic acid	FABP3
caprylic acid	FABP2
caprylic acid	FABP1
caprylic acid	ALDH3A2
caprylic acid	FKBP1A
caprylic acid	FURIN
caprylic acid	FURIN
caprylic acid	GM2A
caprylic acid	MBOAT4
caprylic acid	PAEP
caprylic acid	GLTP
caprylic acid	GLTP
caprylic acid	GLTP
caprylic acid	PLA2G2D
caprylic acid	GUCA1A
caprylic acid	HNF4A
caprylic acid	HNF4A
caprylic acid	HNF4G
caprylic acid	EP300
caprylic acid	HNF4A
caprylic acid	HNF4G
caprylic acid	INS
caprylic acid	CXCL8
caprylic acid	IAH1
caprylic acid	ARF1
caprylic acid	ARF1
caprylic acid	ARF6
caprylic acid	ACSL1
caprylic acid	ACSL3
caprylic acid	ACSL4
caprylic acid	ACSL5
caprylic acid	ACSL6
caprylic acid	ACSBG1
caprylic acid	LY96
caprylic acid	LY96
caprylic acid	ALB
caprylic acid	ALDH1B1
caprylic acid	ABCB1
caprylic acid	PMP2

caprylic acid	NCOA1
caprylic acid	PPT1
caprylic acid	FKBP1A
caprylic acid	FKBP1A
caprylic acid	PPARA
caprylic acid	PPARG
caprylic acid	ABCB1
caprylic acid	PLA2G2C
caprylic acid	PLA2G1B
caprylic acid	PLA2G2A
caprylic acid	PVR
caprylic acid	GM2A
quercetin	CYP24A1
quercetin	SCMH1
quercetin	GALNT1
quercetin	GALNT10
quercetin	GALNT3
quercetin	LSMEM1
quercetin	FIG4
quercetin	PTBP1
quercetin	PTBP2
quercetin	CLP1
quercetin	POPDC2
quercetin	KCNU1
quercetin	PIK3CG
quercetin	TRMT61B
quercetin	POU2AF1
quercetin	PQLC3
quercetin	PRDM10
quercetin	ARL6IP5
quercetin	PBX3
quercetin	NR1I2
quercetin	LMNA
quercetin	PRELID2
quercetin	RABAC1
quercetin	PRKRIP1
quercetin	DDX20
quercetin	DDX58
quercetin	SCNN1A
quercetin	DHX40
quercetin	MTHFD2L
quercetin	ATP13A1
quercetin	DIMT1
quercetin	APOBEC3B
quercetin	HECTD2
quercetin	SLC18B1
quercetin	TRIP12
quercetin	C14orf1
quercetin	GPX8
quercetin	METTL18
quercetin	GPR160
quercetin	USP35
quercetin	MAOA
quercetin	GPR83
quercetin	HELZ
quercetin	AMDHD1
quercetin	LMBRD1
quercetin	C7orf60
quercetin	TARBP1

quercetin	NAT8
quercetin	IFI27L1
quercetin	ZDHHC23
quercetin	MAOB
quercetin	ATP8B1
quercetin	ATP9A
quercetin	SLC7A6OS
quercetin	RBM19
quercetin	RBM23
quercetin	CPVL
quercetin	MARCH3
quercetin	OSGEPL1
quercetin	MN1
quercetin	XPNPEP3
quercetin	PCOLCE
quercetin	PCOLCE2
quercetin	PLOD2
quercetin	ACY1
quercetin	EGF
quercetin	EREG
quercetin	AMT
quercetin	PAQR3
quercetin	PAQR9
quercetin	ANKH
quercetin	HBEGF
quercetin	PCNA
quercetin	PA2G4
quercetin	PROSC
quercetin	PSRC1
quercetin	PRAP1
quercetin	AMMECR1
quercetin	PRR15
quercetin	PRR7
quercetin	PRRT1
quercetin	PSTPIP1
quercetin	P4HA1
quercetin	AMPD2
quercetin	TMEM159
quercetin	PROM1
quercetin	AMPD3
quercetin	PCCA
quercetin	PCSK5
quercetin	PCSK9
quercetin	PROX1
quercetin	PTGES3
quercetin	PTGER3
quercetin	PTGFRN
quercetin	PTGS1
quercetin	PTGS2
quercetin	PTGR1
quercetin	PTGR2
quercetin	PSCA
quercetin	KLK3
quercetin	ACPP
quercetin	ADRM1
quercetin	PSME4
quercetin	PSMG1
quercetin	PSMG4
quercetin	MLLT3

quercetin	AMN1
quercetin	PRMT5
quercetin	EIF2C2
quercetin	APBA3
quercetin	ASTE1
quercetin	BSN
quercetin	BEX1
quercetin	BTG1
quercetin	BTG2
quercetin	C3orf33
quercetin	CASC5
quercetin	RUNX1T1
quercetin	APBB2
quercetin	KIAA1524
quercetin	CYR61
quercetin	DBF4
quercetin	DEK
quercetin	DEPP
quercetin	P4HB
quercetin	PDIA3
quercetin	TMX3
quercetin	DOPEY1
quercetin	DPY30
quercetin	MRPS31
quercetin	ECT2
quercetin	EFR3A
quercetin	ENAH
quercetin	LMAN1
quercetin	FADD
quercetin	FAM102B
quercetin	FAM110B
quercetin	APLP1
quercetin	FAM117A
quercetin	FAM13A
quercetin	FAM13B
quercetin	FAM150B
quercetin	ROBO1
quercetin	FAM162A
quercetin	FAM171A1
quercetin	FAM177A1
quercetin	FAM193A
quercetin	FAM198B
quercetin	FAM19A4
quercetin	FAM19A5
quercetin	FAM212B
quercetin	C21orf59
quercetin	FAM3B
quercetin	FAM46A
quercetin	FAM46B
quercetin	FAM46C
quercetin	FAM50A
quercetin	FAM53C
quercetin	ANAPC10
quercetin	FAM98A
quercetin	FAM98B
quercetin	RPGRIP1L
quercetin	FEM1C
quercetin	C6orf48
quercetin	HEXIM2

quercetin	HOOK1
quercetin	HOOK2
quercetin	HOOK3
quercetin	INTU
quercetin	JAG1
quercetin	KHNYN
quercetin	WWC1
quercetin	PRKCA
quercetin	ANAPC4
quercetin	PRKCB
quercetin	PRKCI
quercetin	PRKCQ
quercetin	ELOB
quercetin	PKDCC
quercetin	DNAAF2
quercetin	KTI12
quercetin	TMBIM4
quercetin	LIN28B
quercetin	AR
quercetin	OIP5
quercetin	KIAA0753
quercetin	NKD2
quercetin	NDRG1
quercetin	NDRG2
quercetin	NIPSNAP3A
quercetin	METTL21A
quercetin	FAM114A1
quercetin	NTAN1
quercetin	OSR2
quercetin	PTCH1
quercetin	PELO
quercetin	PPP1R15A
quercetin	PPP1R16A
quercetin	PPP1R1A
quercetin	PPP1R3C
quercetin	PPP1R3D
quercetin	PPM1D
quercetin	PPM1H
quercetin	PPM1M
quercetin	PBRM1
quercetin	AIG1
quercetin	PRUNE
quercetin	BBS9
quercetin	RIPPLY3
quercetin	SSUH2
quercetin	S100A1
quercetin	S100A11
quercetin	S100A13
quercetin	S100A16
quercetin	S100A2
quercetin	S100A3
quercetin	S100A6
quercetin	S100P
quercetin	ANG
quercetin	SMG9
quercetin	MRPS6
quercetin	SPRY1
quercetin	TIMELESS
quercetin	AMOTL2

quercetin	SEC24D
quercetin	TEK
quercetin	TTYH3
quercetin	UNC50
quercetin	WNT11
quercetin	WWC2
quercetin	TIFA
quercetin	SPACIA2
quercetin	YPEL2
quercetin	SERPINA10
quercetin	ZWILCH
quercetin	F2R
quercetin	F2RL1
quercetin	TGM2
quercetin	ANGPTL4
quercetin	SLC44A5
quercetin	LOX
quercetin	METTL21B
quercetin	TESK2
quercetin	METTL10
quercetin	N6AMT2
quercetin	MICAL1
quercetin	MICAL3
quercetin	PTK6
quercetin	TPST2
quercetin	F2
quercetin	MAP3K21
quercetin	ZNF260
quercetin	PCDHB2
quercetin	FOS
quercetin	FRAT1
quercetin	MOS
quercetin	AGT
quercetin	RET
quercetin	SRC
quercetin	TGFA
quercetin	GULP1
quercetin	RAI14
quercetin	PLEKHG4
quercetin	PNP
quercetin	NPEPPS
quercetin	BTBD11
quercetin	HCK
quercetin	PHTF1
quercetin	PHTF2
quercetin	MOGAT1
quercetin	NSUN6
quercetin	HS3ST5
quercetin	ANKMY2
quercetin	UBAP1L
quercetin	RAB42
quercetin	RBM3
quercetin	SLC38A11
quercetin	PUS10
quercetin	C6orf52
quercetin	ZNF552
quercetin	ANKS4B
quercetin	RBP1
quercetin	PYCR2

quercetin	PKM2
quercetin	MOGAT2
quercetin	TP53I3
quercetin	CRYZL1
quercetin	GDI1
quercetin	CHML
quercetin	RABEPK
quercetin	AKT1
quercetin	RINT1
quercetin	RAD51AP1
quercetin	IER3
quercetin	RSAD1
quercetin	RSAD2
quercetin	RAF1
quercetin	RALGDS
quercetin	RGL1
quercetin	RANBP10
quercetin	RANBP6
quercetin	RBCK1
quercetin	RAPGEF2
quercetin	ASB2
quercetin	RASSF1
quercetin	RASA1
quercetin	IQGAP1
quercetin	IQGAP2
quercetin	RAPH1
quercetin	RAB1B
quercetin	RAB20
quercetin	RAB23
quercetin	ASB9
quercetin	RAB28
quercetin	RAB3B
quercetin	RAB40B
quercetin	RAB8B
quercetin	ANKRD10
quercetin	RALA
quercetin	RALB
quercetin	RRAS
quercetin	ANKRD12
quercetin	RCBTB1
quercetin	RAMP1
quercetin	REEP6
quercetin	ERBB2
quercetin	ERBB3
quercetin	RIPK1
quercetin	RIPK2
quercetin	RIPK3
quercetin	RIPK4
quercetin	FLT3
quercetin	PTPRG
quercetin	PTPRH
quercetin	PTPRK
quercetin	PTPRN2
quercetin	ANKRD16
quercetin	PTPRU
quercetin	MGA
quercetin	RGN
quercetin	RGCC
quercetin	RGS1

quercetin	RGS10
quercetin	RGS19
quercetin	RGS2
quercetin	RGS20
quercetin	RGS5
quercetin	RGS7
quercetin	UPF3B
quercetin	RFXAP
quercetin	PPP1R13L
quercetin	RFC3
quercetin	RFC4
quercetin	RMND1
quercetin	DNAJC3
quercetin	RTN4IP1
quercetin	ALDH1A1
quercetin	AOC2
quercetin	RB1
quercetin	RARB
quercetin	RARRES2
quercetin	RXRG
quercetin	GPRC5A
quercetin	RDH12
quercetin	RBP4
quercetin	PEG10
quercetin	ASPRV1
quercetin	RECK
quercetin	ARHGDIB
quercetin	ARHGAP11A
quercetin	PCNX4
quercetin	ANKRD37
quercetin	ARHGAP17
quercetin	ARHGAP19
quercetin	ARHGAP27
quercetin	ARHGAP44
quercetin	ARHGEF3
quercetin	ROCK1
quercetin	ROCK2
quercetin	RHBDD2
quercetin	RHBDD3
quercetin	RHBDL2
quercetin	RHOBTB1
quercetin	RHOBTB3
quercetin	RND1
quercetin	RHOC
quercetin	RHOF
quercetin	RHOU
quercetin	ANKRD5
quercetin	RBKS
quercetin	DROSHA
quercetin	RNASEH2A
quercetin	RPP38
quercetin	RPP40
quercetin	HACL1
quercetin	RAVER2
quercetin	RRM1
quercetin	RRM2
quercetin	RRM2B
quercetin	RSL1D1
quercetin	RPS6KA1

quercetin	RPS6KA3
quercetin	RRP7A
quercetin	WDR12
quercetin	RRS1
quercetin	RRBP1
quercetin	NQO2
quercetin	RPE
quercetin	RILPL2
quercetin	RNFT1
quercetin	RNF141
quercetin	RNF24
quercetin	C4orf48
quercetin	RTCA
quercetin	ELL2
quercetin	RPAP1
quercetin	RBMS1
quercetin	RBMS2
quercetin	RBM12
quercetin	UGT8
quercetin	RBM24
quercetin	RBM38
quercetin	RBM4
quercetin	RBM45
quercetin	RBM4B
quercetin	ANKRA2
quercetin	ZFP69
quercetin	RUFY1
quercetin	ANK2
quercetin	FBXO30
quercetin	RUNDC3B
quercetin	RUNX2
quercetin	RUVBL2
quercetin	RUVBL2
quercetin	RUVBL2
quercetin	RUVBL2
quercetin	RWDD2A
quercetin	RWDD2B
quercetin	NUDT14
quercetin	ANXA1
quercetin	SCAPER
quercetin	SACS
quercetin	SALL1
quercetin	SASH1
quercetin	SAMHD1
quercetin	ATP2A1
quercetin	SAFB
quercetin	SAFB2
quercetin	ANXA2
quercetin	STIL
quercetin	SCFD2
quercetin	SFRP1
quercetin	FRZB
quercetin	ANXA6
quercetin	PTTG1
quercetin	BSCL2
quercetin	SELENBP1
quercetin	SEMA3C
quercetin	SEMA3F
quercetin	SEMA3G

quercetin	SEMA4G
quercetin	SENP7
quercetin	SQSTM1
quercetin	LACTB
quercetin	SERINC2
quercetin	METTL12
quercetin	SPTLC3
quercetin	HPN
quercetin	RSRC1
quercetin	SRSF1
quercetin	SRSF3
quercetin	AGR2
quercetin	SRSF7
quercetin	STK17B
quercetin	STYX
quercetin	PIM1
quercetin	STK16
quercetin	STK17A
quercetin	STK17B
quercetin	STK38
quercetin	STK40
quercetin	ATR
quercetin	CHEK2
quercetin	PRKD1
quercetin	PRKD3
quercetin	ANTXR2
quercetin	MASTL
quercetin	UHMK1
quercetin	CDC42BPB
quercetin	MTOR
quercetin	PKN1
quercetin	NEK1
quercetin	NEK2
quercetin	NEK4
quercetin	NIM1K
quercetin	NLK
quercetin	TAP1
quercetin	PDIK1L
quercetin	PIM1
quercetin	PIM2
quercetin	PIM3
quercetin	PLK1
quercetin	PLK2
quercetin	PLK3
quercetin	PRPF4B
quercetin	SGK1
quercetin	SMG1
quercetin	STK11
quercetin	TAOK3
quercetin	TBK1
quercetin	TLK1
quercetin	VRK1
quercetin	VRK2
quercetin	ERN1
quercetin	ERN2
quercetin	PPP2R1B
quercetin	PPP2R4
quercetin	PPP2R3C
quercetin	PPP3CA

quercetin	SERPINC1
quercetin	PPP4R4
quercetin	PPP6R2
quercetin	SERPINB3
quercetin	SERPINB9
quercetin	SERTAD1
quercetin	ALB
quercetin	SDPR
quercetin	PON1
quercetin	PON2
quercetin	SESN1
quercetin	SESN2
quercetin	SESN3
quercetin	SETD5
quercetin	SCML2
quercetin	SHBG
quercetin	SHBG
quercetin	AP1M2
quercetin	SHBG
quercetin	SH2D5
quercetin	AP1S1
quercetin	MPHOSPH9
quercetin	SH2B3
quercetin	SH3PXD2A
quercetin	SH3BGR
quercetin	SH3BGRL
quercetin	SH3BGRL2
quercetin	SH3BGRL3
quercetin	SH3BP2
quercetin	SH3D19
quercetin	SH3YL1
quercetin	ACADSB
quercetin	SDR42E1
quercetin	SGO2
quercetin	LRP5L
quercetin	STAT1
quercetin	SOBP
quercetin	AP3M2
quercetin	SSBP1
quercetin	PDS5B
quercetin	MIS18BP1
quercetin	SRGAP3
quercetin	ZNF581
quercetin	UTP20
quercetin	EFCAB11
quercetin	BHMT2
quercetin	SMTN
quercetin	AP5M1
quercetin	SNAPC4
quercetin	SLC6A12
quercetin	SLC6A8
quercetin	SLC6A11
quercetin	SLC4A7
quercetin	SLC4A11
quercetin	SCLT1
quercetin	SCN1A
quercetin	SCN9A
quercetin	A1CF
quercetin	SLC9A6

quercetin	SLC9A8
quercetin	SLC5A3
quercetin	ATP1A1
quercetin	NKAIN1
quercetin	ATP1B3
quercetin	FXYD2
quercetin	APOA5
quercetin	GUCD1
quercetin	SLC38A1
quercetin	SLC38A2
quercetin	SLC38A4
quercetin	SLC38A9
quercetin	APOB
quercetin	SLC17A2
quercetin	SLC20A1
quercetin	SLC6A4
quercetin	APOC2
quercetin	SLC12A2
quercetin	SLC12A4
quercetin	SLC13A5
quercetin	SLC16A1
quercetin	SLC16A7
quercetin	SLC17A9
quercetin	SLC2A1
quercetin	APOE
quercetin	SLC2A10
quercetin	SLC2A14
quercetin	SLC2A2
quercetin	SLC2A3
quercetin	SLC2A4
quercetin	SLC2A5
quercetin	APOF
quercetin	SLC2A7
quercetin	SLC2A8
quercetin	SLC2A9
quercetin	APOLD1
quercetin	SLC22A15
quercetin	GBE1
quercetin	SLC22A3
quercetin	SLC22A5
quercetin	SLC22A9
quercetin	SLC23A1
quercetin	SLC25A39
quercetin	APOM
quercetin	SLC25A43
quercetin	SLC25A45
quercetin	SLC25A46
quercetin	SLC35F5
quercetin	SLC35G2
quercetin	SLC40A1
quercetin	SLC41A1
quercetin	SLC41A2
quercetin	BCL2L14
quercetin	SLCO2B1
quercetin	SLCO2B1
quercetin	SLCO4A1
quercetin	SLCO4C1
quercetin	BAX
quercetin	SLCO2B1

quercetin	HSD3B7
quercetin	SOS1
quercetin	SORBS2
quercetin	BCL2
quercetin	CDCA5
quercetin	SNX14
quercetin	SNX24
quercetin	SNX7
quercetin	SNX8
quercetin	OBFC2A
quercetin	SPAST
quercetin	AEN
quercetin	SPTBN1
quercetin	AIFM1
quercetin	SPA17
quercetin	SPAG1
quercetin	SPAG16
quercetin	SPAG5
quercetin	STRBP
quercetin	SPATA2L
quercetin	SMOX
quercetin	SKP2
quercetin	TP53BP2
quercetin	SASS6
quercetin	SPIN3
quercetin	SF3A2
quercetin	SF3B3
quercetin	SF3B4
quercetin	APAF1
quercetin	SPOCD1
quercetin	SPSB1
quercetin	SQLE
quercetin	FDFT1
quercetin	SKAP2
quercetin	CTTN
quercetin	STC1
quercetin	STC2
quercetin	STBD1
quercetin	STARD13
quercetin	STARD9
quercetin	STMN1
quercetin	AQP11
quercetin	SAMD9
quercetin	SOAT1
quercetin	SOAT2
quercetin	SREBF1
quercetin	NSDHL
quercetin	AQP3
quercetin	STK17B
quercetin	SDF2L1
quercetin	SMAP1
quercetin	MMP3
quercetin	MMP10
quercetin	MMP11
quercetin	SMC2
quercetin	SMC3
quercetin	SMC6
quercetin	ADH6
quercetin	TOR1AIP1

quercetin	FPGT
quercetin	C11orf80
quercetin	HSD3B1
quercetin	ALOX12B
quercetin	ZNF788
quercetin	VEGFA
quercetin	ALOX12
quercetin	CYP51A1
quercetin	SKIDA1
quercetin	ALOX15
quercetin	ALOX15B
quercetin	LMO7
quercetin	MFN1
quercetin	ALOX5
quercetin	GCKR
quercetin	STAU2
quercetin	CYP4F12
quercetin	HSD3B2
quercetin	FNDC5
quercetin	FDXR
quercetin	ALOX5AP
quercetin	TTLL3
quercetin	OTUD6B
quercetin	ATXN3
quercetin	SLC3A2
quercetin	GPRC5C
quercetin	PPM1K
quercetin	EXOC5
quercetin	ZFP41
quercetin	NCEH1
quercetin	TAB3
quercetin	RGMB
quercetin	MROH8
quercetin	MYD88
quercetin	EFCAB8
quercetin	N/A
quercetin	ECD
quercetin	HPR
quercetin	BAK1
quercetin	ANKHD1
quercetin	IRAK4
quercetin	MUC15
quercetin	SDHC
quercetin	ALDH5A1
quercetin	SUCLG2
quercetin	ASAP2
quercetin	SLC37A2
quercetin	SLC26A2
quercetin	QSOX1
quercetin	SQRDL
quercetin	SUOX
quercetin	SULT1A1
quercetin	SULT1A2
quercetin	SULT1C2
quercetin	ARG1
quercetin	SKIV2L2
quercetin	ARG2
quercetin	SOD1
quercetin	SAPCD2

quercetin	SOCS4
quercetin	SOCS6
quercetin	ST7L
quercetin	SUSD3
quercetin	SRPX
quercetin	SRPX2
quercetin	VAT1
quercetin	SYNGR3
quercetin	SYCE2
quercetin	SYTL1
quercetin	SYTL4
quercetin	MATK
quercetin	SYNC
quercetin	SDC2
quercetin	ASS1
quercetin	SDC4
quercetin	SYBU
quercetin	STX16
quercetin	STX1A
quercetin	STX3
quercetin	STX5
quercetin	STXBP5
quercetin	TAC4
quercetin	TAF5L
quercetin	WRB
quercetin	TNKS
quercetin	SMIM3
quercetin	TAF1A
quercetin	ARVCF
quercetin	TBCK
quercetin	FBXO11
quercetin	TBC1D7
quercetin	TBC1D8
quercetin	TBC1D8B
quercetin	TBC1D9
quercetin	TBX3
quercetin	FAM209B
quercetin	CD7
quercetin	TCTA
quercetin	CD3D
quercetin	TCP11L1
quercetin	TCTEX1D2
quercetin	TSHZ1
quercetin	AKR1E2
quercetin	TEP1
quercetin	TERT
quercetin	ARMCX5
quercetin	TNS1
quercetin	TEX19
quercetin	DDC
quercetin	TEX9
quercetin	TSSK1B
quercetin	TSSK2
quercetin	TSSK3
quercetin	TSSK4
quercetin	TSPYL2
quercetin	TSPYL4
quercetin	FAM208A
quercetin	ASPSCR1

quercetin	ARRDC2
quercetin	TSPAN3
quercetin	TSPAN4
quercetin	TSPAN5
quercetin	TSPAN8
quercetin	ARRDC3
quercetin	TTC14
quercetin	TTC17
quercetin	TTC27
quercetin	TTC30B
quercetin	TTC33
quercetin	TTC39C
quercetin	MRPL13
quercetin	TTC7B
quercetin	TGFBR2
quercetin	THAP10
quercetin	THAP2
quercetin	SLC19A3
quercetin	CRYM
quercetin	TXNDC15
quercetin	TXNDC16
quercetin	AHR
quercetin	TXNRD1
quercetin	TST
quercetin	THOC1
quercetin	THNSL1
quercetin	CDKAL1
quercetin	THBD
quercetin	ARNT
quercetin	THBS1
quercetin	THBS2
quercetin	ARNTL
quercetin	TYMS
quercetin	THYN1
quercetin	TMSB10
quercetin	ARNTL2
quercetin	AHRR
quercetin	SERPINA7
quercetin	TIGD1
quercetin	TIGD2
quercetin	TJP1
quercetin	AHR
quercetin	TJP2
quercetin	TIPIN
quercetin	TICAM1
quercetin	F3
quercetin	TFPI
quercetin	TFPI2
quercetin	PLAT
quercetin	TM2D3
quercetin	TRAF4
quercetin	TLR1
quercetin	TLR10
quercetin	TLR2
quercetin	TLR4
quercetin	TLR6
quercetin	TMEFF1
quercetin	TONSL
quercetin	TOR2A

quercetin	TP53RK
quercetin	TTMP
quercetin	ARSE
quercetin	TRAPPC10
quercetin	TRAPPC2
quercetin	TRAPPC6A
quercetin	ARSG
quercetin	TALDO1
quercetin	TCEAL8
quercetin	TEFM
quercetin	TCF12
quercetin	TFAM
quercetin	JUN
quercetin	BTF3L4
quercetin	CEBPB
quercetin	TFDP2
quercetin	E2F1
quercetin	E2F2
quercetin	E2F3
quercetin	E2F5
quercetin	E2F7
quercetin	ASGR1
quercetin	GATA6
quercetin	HES1
quercetin	HES4
quercetin	HIVEP2
quercetin	JUNB
quercetin	JUND
quercetin	MAFF
quercetin	MAFG
quercetin	RELA
quercetin	RELB
quercetin	SOX30
quercetin	SP1
quercetin	SP5
quercetin	GTF2E2
quercetin	TAF3
quercetin	TAF5
quercetin	TAF9
quercetin	TAF9B
quercetin	TRIM24
quercetin	MTERF2
quercetin	MYB
quercetin	TADA1
quercetin	TFRC
quercetin	TRRAP
quercetin	TRA2A
quercetin	TGFB1
quercetin	TGFB1I1
quercetin	TAGLN
quercetin	ASPHD1
quercetin	TRPM7
quercetin	TRPV1
quercetin	TRPV2
quercetin	DARS
quercetin	TKT
quercetin	GUF1
quercetin	EIF2B2
quercetin	MTIF2

quercetin	MTIF3
quercetin	TRAM1L1
quercetin	SSR3
quercetin	TM4SF1
quercetin	TM7SF3
quercetin	TMC7
quercetin	ASPH
quercetin	TMED6
quercetin	GPNMB
quercetin	FHIT
quercetin	TMPRSS11D
quercetin	TMPRSS11F
quercetin	TMEM117
quercetin	TMEM126A
quercetin	TMEM128
quercetin	KIAA0922
quercetin	TMEM135
quercetin	TMEM138
quercetin	PEA15
quercetin	TMEM139
quercetin	TMEM140
quercetin	TMEM14A
quercetin	TMEM165
quercetin	TMEM168
quercetin	TMEM170B
quercetin	TMEM187
quercetin	TMEM19
quercetin	TMEM192
quercetin	TMEM2
quercetin	TMEM222
quercetin	C17orf75
quercetin	TMEM41B
quercetin	TMEM45A
quercetin	TMEM54
quercetin	TMEM60
quercetin	TMEM64
quercetin	TMEM65
quercetin	TMEM68
quercetin	TMEM80
quercetin	TMEM9B
quercetin	C1orf162
quercetin	C9orf91
quercetin	ATL1
quercetin	TTR
quercetin	ATL2
quercetin	TFF1
quercetin	TRIB1
quercetin	TRIB2
quercetin	TRIB3
quercetin	SLC25A1
quercetin	TREM2
quercetin	TRIOBP
quercetin	TPI1
quercetin	TRIM47
quercetin	ZC2HC1C
quercetin	TRIM59
quercetin	TPP1
quercetin	ATP5MC2
quercetin	ZFP36

quercetin	TRMT11
quercetin	TYW3
quercetin	TSEN15
quercetin	TPBG
quercetin	TPM1
quercetin	TPM4
quercetin	TNNC1
quercetin	TNNI2
quercetin	ATP5F1A
quercetin	CYP1B1
quercetin	MRPL20
quercetin	ATP5B
quercetin	ATP5F1B
quercetin	TSC2
quercetin	TUBA1A
quercetin	TUBB
quercetin	TUBB1
quercetin	TUBB2B
quercetin	TUBB6
quercetin	TUBD1
quercetin	TDRD3
quercetin	TDRD7
quercetin	TUFT1
quercetin	TNF
quercetin	TNFAIP8
quercetin	TNFSF10
quercetin	TNFSF4
quercetin	FASLG
quercetin	TNFRSF10A
quercetin	TNFRSF10B
quercetin	TNFRSF10D
quercetin	HSPE1
quercetin	TNFRSF11A
quercetin	TNFRSF11B
quercetin	TNFRSF12A
quercetin	TNFRSF14
quercetin	TNFRSF19
quercetin	TNFRSF1B
quercetin	TNFRSF21
quercetin	EDA2R
quercetin	FAS
quercetin	ATP5F1C
quercetin	TP63
quercetin	TP53INP1
quercetin	TP73
quercetin	TSG101
quercetin	DIO1
quercetin	AGTR1
quercetin	TYR
quercetin	HCK
quercetin	JAK1
quercetin	ABL1
quercetin	ABL2
quercetin	BLK
quercetin	BTK
quercetin	CSK
quercetin	FGR
quercetin	FYN
quercetin	HCK

quercetin	ITK
quercetin	JAK1
quercetin	JAK2
quercetin	JAK3
quercetin	LCK
quercetin	LYN
quercetin	MERTK
quercetin	MRPL23
quercetin	TIE1
quercetin	TYRO3
quercetin	AXL
quercetin	RYK
quercetin	SRMS
quercetin	STYK1
quercetin	SYK
quercetin	TEC
quercetin	ROR1
quercetin	TXK
quercetin	YES1
quercetin	ZAP70
quercetin	YARS2
quercetin	ABCA1
quercetin	TDP2
quercetin	IMP3
quercetin	UTP18
quercetin	SART1
quercetin	LSM4
quercetin	ABCA12
quercetin	USP13
quercetin	ABCA5
quercetin	USP4
quercetin	USP43
quercetin	USP46
quercetin	UCHL3
quercetin	ABCA7
quercetin	UBD
quercetin	UFD1L
quercetin	OTUB2
quercetin	UBASH3B
quercetin	UBAP2
quercetin	UBE2E2
quercetin	UBE2L3
quercetin	UBE2Q2
quercetin	UFM1
quercetin	UBA1
quercetin	UBA5
quercetin	UBL3
quercetin	ISG15
quercetin	ATG3
quercetin	UBE3B
quercetin	UGT3A1
quercetin	SLC35A2
quercetin	B3GNTL1
quercetin	UXS1
quercetin	SLC35D1
quercetin	UGT3A1
quercetin	UGT1A1
quercetin	UGT1A10
quercetin	UGT1A3

quercetin	UGT1A4
quercetin	UGT1A5
quercetin	UGT1A6
quercetin	UGT1A7
quercetin	UGT1A8
quercetin	UGT1A9
quercetin	UGT2A1
quercetin	UGT2A3
quercetin	UGT2B10
quercetin	UGT2B11
quercetin	UGT2B15
quercetin	UGT2B17
quercetin	UGT2B28
quercetin	UGT2B4
quercetin	UGT2B7
quercetin	UGT3A1
quercetin	UGT3A2
quercetin	ALG13
quercetin	ALG14
quercetin	UAP1L1
quercetin	UFSP2
quercetin	CMPK2
quercetin	UNC5CL
quercetin	MFSD11
quercetin	ABCB1
quercetin	C11orf52
quercetin	C11orf96
quercetin	C12orf60
quercetin	C14orf118
quercetin	C14orf28
quercetin	PRRC2B
quercetin	C16orf45
quercetin	CEL
quercetin	C17orf62
quercetin	ZNF225
quercetin	ABCD3
quercetin	C19orf18
quercetin	C1orf115
quercetin	SAP18
quercetin	C1orf64
quercetin	C22orf25
quercetin	C2orf72
quercetin	C2orf82
quercetin	C3orf38
quercetin	C3orf62
quercetin	C4orf3
quercetin	C5orf34
quercetin	C6orf226
quercetin	C7orf50
quercetin	C7orf58
quercetin	C8orf4
quercetin	C9orf152
quercetin	C9orf85
quercetin	ABCG2
quercetin	KIAA0528
quercetin	KIAA0556
quercetin	KIAA0895L
quercetin	KIAA1211
quercetin	MYO1B

quercetin	ABCG5
quercetin	MYO5A
quercetin	MYO7A
quercetin	ABCG8
quercetin	URB2
quercetin	C16orf58
quercetin	C19orf25
quercetin	C17orf58
quercetin	KIAA0907
quercetin	ABCC1
quercetin	C20orf94
quercetin	C1orf216
quercetin	C2orf76
quercetin	C16orf87
quercetin	C7orf55
quercetin	ABCC1
quercetin	C4orf27
quercetin	ABCC1
quercetin	ABCC1
quercetin	ABCB1
quercetin	UCN
quercetin	PLAU
quercetin	UPK3A
quercetin	ABCB1
quercetin	UROD
quercetin	USP6NL
quercetin	UGP2
quercetin	VPS16
quercetin	VPS54
quercetin	VPS36
quercetin	VANGL1
quercetin	ABCG2
quercetin	VCAM1
quercetin	VEGFA
quercetin	FIGF
quercetin	FLT1
quercetin	ABCG2
quercetin	KDR
quercetin	FLT4
quercetin	MPC2
quercetin	VIPR1
quercetin	VASN
quercetin	SLC27A2
quercetin	ACADVL
quercetin	VLDLR
quercetin	GOLT1A
quercetin	SFT2D2
quercetin	CLPX
quercetin	SEC22A
quercetin	PIF1
quercetin	VIL1
quercetin	VSNL1
quercetin	RECQL
quercetin	PROC
quercetin	PROS1
quercetin	VTN
quercetin	VDAC1
quercetin	VHL
quercetin	VSIG10L

quercetin	VSIG2
quercetin	ATP6V0B
quercetin	ATP6V1C1
quercetin	DDX39A
quercetin	ATP6V0E2
quercetin	KIAA0196
quercetin	WDFY1
quercetin	WSB1
quercetin	WDSUB1
quercetin	PIEZO2
quercetin	WDR34
quercetin	WDR41
quercetin	WDR44
quercetin	WDR47
quercetin	WDR53
quercetin	WDR54
quercetin	WDR5B
quercetin	WDR61
quercetin	WDR66
quercetin	WDR7
quercetin	MIOS
quercetin	DHX29
quercetin	WBP1L
quercetin	WWTR1
quercetin	XDH
quercetin	XBP1
quercetin	YAP1
quercetin	YRDC
quercetin	ZBTB10
quercetin	ZBTB2
quercetin	ZBTB38
quercetin	KCNJ10
quercetin	FBXO31
quercetin	ZSCAN21
quercetin	ZSCAN4
quercetin	ZBED3
quercetin	ZBED5
quercetin	HEMK2
quercetin	C13orf34
quercetin	ZC3H6
quercetin	ZC3H7A
quercetin	KCNJ14
quercetin	MTFR2
quercetin	ZCCHC2
quercetin	ZCCHC7
quercetin	ZFYVE1
quercetin	ZFYVE16
quercetin	ZMAT3
quercetin	ZMYM6
quercetin	ZNF112
quercetin	ZNF117
quercetin	ZNF138
quercetin	ZFP161
quercetin	ZNF165
quercetin	EID3
quercetin	ZNF184
quercetin	ZNF189
quercetin	NPR3
quercetin	ZNF217

quercetin	ZNF227
quercetin	ZNF23
quercetin	ZNF248
quercetin	RPL18A
quercetin	ARID2
quercetin	ZNF280A
quercetin	SMIM19
quercetin	ZNF30
quercetin	ZNF337
quercetin	ZNF33B
quercetin	ZFP36L1
quercetin	ZNF362
quercetin	ZFP37
quercetin	ZNF385B
quercetin	SHPRH
quercetin	ZNF395
quercetin	AURKA
quercetin	ZNF439
quercetin	ZNF440
quercetin	ZNF441
quercetin	PTCHD4
quercetin	AURKB
quercetin	ZNF513
quercetin	AAED1
quercetin	FAM117B
quercetin	ZNF555
quercetin	ZNF557
quercetin	ZNF559
quercetin	ZNF561
quercetin	ZNF565
quercetin	ZNF57
quercetin	ZNF573
quercetin	ARHGEF28
quercetin	MRPL39
quercetin	ATG16L2
quercetin	ZNF580
quercetin	ATG2A
quercetin	ZNF594
quercetin	FAM193B
quercetin	ZNF600
quercetin	ZNF608
quercetin	ZNF624
quercetin	TCF19
quercetin	TMEM156
quercetin	ZNF639
quercetin	ZNF652
quercetin	FAM209A
quercetin	REEP3
quercetin	ZNF684
quercetin	AXIN2
quercetin	ZNF700
quercetin	ZNF708
quercetin	ZNF711
quercetin	ZFP69B
quercetin	ZNF789
quercetin	ZNF79
quercetin	SLC7A9
quercetin	ZNF84
quercetin	PLAG1

quercetin	PLAGL1
quercetin	RLF
quercetin	SNAI1
quercetin	C6orf1
quercetin	ZIC2
quercetin	ZFPL1
quercetin	ZSWIM5
quercetin	ZSWIM6
quercetin	ZFX
quercetin	ZXDB
quercetin	ZHX2
quercetin	SLC30A1
quercetin	SLC30A10
quercetin	SLC30A2
quercetin	SLC30A3
quercetin	SLC30A4
quercetin	SLC30A5
quercetin	SLC30A6
quercetin	SLC30A7
quercetin	SLC30A8
quercetin	SLC39A10
quercetin	SLC39A11
quercetin	SLC39A14
quercetin	SLC39A4
quercetin	ZG16B
quercetin	BIRC5
quercetin	NOP9
quercetin	BAG2
quercetin	BAG4
quercetin	EPB41L1
quercetin	EPB41L5
quercetin	BBS12
quercetin	BBS2
quercetin	BBS4
quercetin	BBS5
quercetin	BCAM
quercetin	BIVM
quercetin	BSG
quercetin	MRPL47
quercetin	MRPL49
quercetin	MRPL50
quercetin	BAD
quercetin	BNIP3
quercetin	BNIP3L
quercetin	BIK
quercetin	BCL2L1
quercetin	BCL2L2
quercetin	BOK
quercetin	BCL2A1
quercetin	BCOR
quercetin	ACTB
quercetin	B3GALTL
quercetin	RFNG
quercetin	B4GALNT1
quercetin	HMGCR
quercetin	B4GALT4
quercetin	SNTB1
quercetin	APOH
quercetin	ARRB1

quercetin	ACTR1B
quercetin	CHN2
quercetin	PTPLB
quercetin	CRYGS
quercetin	ST6GAL1
quercetin	HEXB
quercetin	BHMT
quercetin	LACTB2
quercetin	BACE1
quercetin	NAPB
quercetin	BDH2
quercetin	BFAR
quercetin	PNKP
quercetin	NR1H4
quercetin	HIBCH
quercetin	BAAT
quercetin	SULT2A1
quercetin	BLOC1S2
quercetin	ACAA2
quercetin	BLM
quercetin	BAMBI
quercetin	BMP1
quercetin	BMP2
quercetin	BMP4
quercetin	BMP8A
quercetin	BDNF
quercetin	BCAT1
quercetin	FAM175A
quercetin	BCR
quercetin	BCAR1
quercetin	BCAR3
quercetin	GTF2H4
quercetin	ABCG2
quercetin	BRCA1
quercetin	BRCA2
quercetin	AKR1D1
quercetin	ARFGEF1
quercetin	ARFGEF3
quercetin	BAZ1A
quercetin	BAZ2B
quercetin	BRDT
quercetin	BRD2
quercetin	BRD7
quercetin	KCTD13
quercetin	BTBD2
quercetin	KCTD14
quercetin	BCKDK
quercetin	KCTD6
quercetin	C1GALT1C1
quercetin	C2CD4A
quercetin	C2CD2
quercetin	C4BPA
quercetin	C4BPB
quercetin	C5AR1
quercetin	ZMPSTE24
quercetin	CAD
quercetin	CDH1
quercetin	CDH11
quercetin	CDH2

quercetin	ZBTB21
quercetin	CAMK1
quercetin	CAMK2B
quercetin	SLC25A12
quercetin	SLC25A24
quercetin	CADPS2
quercetin	CACYBP
quercetin	RPS23
quercetin	CLGN
quercetin	CLMN
quercetin	CAMTA2
quercetin	CAMKMT
quercetin	CAMSAP3
quercetin	CAPN15
quercetin	CAPN2
quercetin	TRIAP1
quercetin	PDE4C
quercetin	ABCC2
quercetin	FAU
quercetin	CLIP2
quercetin	EVA1B
quercetin	CAPRIN2
quercetin	CHST13
quercetin	CHST15
quercetin	CHST3
quercetin	CA2
quercetin	CBR1
quercetin	CBR4
quercetin	N/A
quercetin	CMBL
quercetin	CPB2
quercetin	CPN1
quercetin	CPN2
quercetin	CPQ
quercetin	INTS13
quercetin	CEACAM5
quercetin	CRLS1
quercetin	CPT1A
quercetin	CPT1B
quercetin	CPT2
quercetin	CARNMT1
quercetin	RAPSN
quercetin	CASD1
quercetin	CSNK2A1
quercetin	CSNK2B
quercetin	CSNK2A1
quercetin	CSNK2A2
quercetin	CSNK2B
quercetin	CFLAR
quercetin	CASP1
quercetin	CASP3
quercetin	CASP6
quercetin	CASP8
quercetin	CASP9
quercetin	CAT
quercetin	CTNNA1
quercetin	CTNNB1
quercetin	CTSB
quercetin	CTSD

quercetin	EPHA6
quercetin	CATSPER3
quercetin	CAV1
quercetin	CITED1
quercetin	CITED2
quercetin	CCR6
quercetin	CCR7
quercetin	CCL2
quercetin	CEBPA
quercetin	CEBPB
quercetin	CNOT8
quercetin	CD109
quercetin	CD151
quercetin	CD302
quercetin	CD40LG
quercetin	SFN
quercetin	PRKAB1
quercetin	CD59
quercetin	CD63
quercetin	CD83
quercetin	CD9
quercetin	CD97
quercetin	CABLES1
quercetin	CDK5RAP2
quercetin	SHBG
quercetin	PGS1
quercetin	CEBPB
quercetin	CADM1
quercetin	ANGPTL8
quercetin	CCPG1
quercetin	CIDEC
quercetin	CIDEB
quercetin	RQCD1
quercetin	NT5E
quercetin	CDC45
quercetin	CDC6
quercetin	CDC7
quercetin	CDC16
quercetin	CDC20
quercetin	CDCA7L
quercetin	CDCA3
quercetin	TP53
quercetin	CNTRL
quercetin	CENPC1
quercetin	CENPF
quercetin	CENPI
quercetin	CENPJ
quercetin	CENPK
quercetin	ITGB3BP
quercetin	MLF1IP
quercetin	STRA13
quercetin	ZW10
quercetin	CENPE
quercetin	TRAPPC11
quercetin	CEP128
quercetin	CEP135
quercetin	CEP170B
quercetin	CEP55
quercetin	CEP68

quercetin	CEP70
quercetin	CEP72
quercetin	CEP78
quercetin	UGCG
quercetin	CERS5
quercetin	CDR2L
quercetin	ALAS1
quercetin	KIAA1551
quercetin	CLN5
quercetin	CP
quercetin	SEC11C
quercetin	MAP3K20
quercetin	CKLF
quercetin	SLC26A3
quercetin	PRKAB2
quercetin	CYP46A1
quercetin	CHKA
quercetin	SLC44A3
quercetin	CSGALNACT2
quercetin	CHPF
quercetin	CHAF1B
quercetin	CBX5
quercetin	CBX6
quercetin	CHD1L
quercetin	CHD2
quercetin	CHTF18
quercetin	CHTF8
quercetin	KIF4A
quercetin	CTRL
quercetin	CGNL1
quercetin	CLYBL
quercetin	CMTM7
quercetin	BHLHE40
quercetin	CLTB
quercetin	HECTD4
quercetin	CLDN1
quercetin	CLDN10
quercetin	CLDN14
quercetin	CLDN4
quercetin	CSTF2
quercetin	SLC35A1
quercetin	CDRT4
quercetin	COTL1
quercetin	F7
quercetin	F10
quercetin	COPE
quercetin	MMAB
quercetin	CFL1
quercetin	STAG1
quercetin	CCHCR1
quercetin	CC2D1B
quercetin	CCDC109B
quercetin	CCDC126
quercetin	CCDC130
quercetin	CCDC138
quercetin	CCDC15
quercetin	CCDC17
quercetin	CCDC62
quercetin	CCDC68

quercetin	CCDC69
quercetin	HSPA1A
quercetin	CCDC81
quercetin	CCDC92
quercetin	FBXO17
quercetin	COIL
quercetin	CIRBP
quercetin	COL1A1
quercetin	COL2A1
quercetin	COL3A1
quercetin	COL7A1
quercetin	COL18A1
quercetin	ESR2
quercetin	CTHRC1
quercetin	COLEC12
quercetin	TMEM27
quercetin	COMMD1
quercetin	COMMD10
quercetin	COMMD8
quercetin	C5
quercetin	CD93
quercetin	C8A
quercetin	C8B
quercetin	C8G
quercetin	CD55
quercetin	CFB
quercetin	CFI
quercetin	OCC1
quercetin	NCAPG
quercetin	NCAPG2
quercetin	CTGF
quercetin	COG5
quercetin	COG6
quercetin	FAM120C
quercetin	COPS4
quercetin	COPS5
quercetin	CPNE2
quercetin	CUTC
quercetin	ATP7A
quercetin	ATP7B
quercetin	CORO2A
quercetin	CORO2B
quercetin	SERPINA6
quercetin	NR2F2
quercetin	RPL15
quercetin	CRP
quercetin	CKB
quercetin	CREBBP
quercetin	CSNK2A1
quercetin	CTC1
quercetin	OBFC1
quercetin	CTPS1
quercetin	CUZD1
quercetin	CUEDC1
quercetin	CUEDC2
quercetin	CELF1
quercetin	CUL4B
quercetin	CXCR4
quercetin	CXCL10

quercetin	CXCL11
quercetin	CXCL16
quercetin	CXCL2
quercetin	ATF3
quercetin	RPL27A
quercetin	CREB3L2
quercetin	CREB3L3
quercetin	CCNA2
quercetin	CDK1
quercetin	CDK2
quercetin	CDK4
quercetin	CDKN2B
quercetin	CDKN2C
quercetin	CDK5R1
quercetin	CDK6
quercetin	CDK8
quercetin	CDK9
quercetin	CDKN1A
quercetin	CDKN1B
quercetin	CDKN1C
quercetin	CDKN2A
quercetin	CDKN3
quercetin	CCNF
quercetin	CCNG2
quercetin	CBS
quercetin	CSTA
quercetin	CSRP2
quercetin	CHORDC1
quercetin	CDO1
quercetin	HSD17B6
quercetin	CRIPT
quercetin	CFTR
quercetin	SLC7A11
quercetin	CDA
quercetin	CYBB
quercetin	CYBA
quercetin	CYB5A
quercetin	CYCS
quercetin	COX11
quercetin	COX19
quercetin	CYP1B1
quercetin	CYP11B1
quercetin	CYP1A1
quercetin	CYP1A2
quercetin	CYP1B1
quercetin	CYP20A1
quercetin	CYP26A1
quercetin	CYP26B1
quercetin	CYP26C1
quercetin	CYP2C8
quercetin	CYP2C9
quercetin	CYP2D6
quercetin	CYP2E1
quercetin	CYP2J2
quercetin	CYP2S1
quercetin	CYP3A4
quercetin	CYP3A5
quercetin	CYP4A11
quercetin	CYP4A22

quercetin	CYP4B1
quercetin	CYP4F11
quercetin	CYP4F22
quercetin	CYP4V2
quercetin	CYP4X1
quercetin	PFKFB3
quercetin	CYP4Z1
quercetin	PFKFB4
quercetin	CYP1B1
quercetin	CYGB
quercetin	PFKP
quercetin	CRLF1
quercetin	CRLF3
quercetin	BMX
quercetin	AGTPBP1
quercetin	PGD
quercetin	DRD4
quercetin	PHGDH
quercetin	NUDT1
quercetin	BDH1
quercetin	APPL2
quercetin	DCUN1D2
quercetin	MMP2
quercetin	DCAF16
quercetin	DCAF5
quercetin	DCAF6
quercetin	DAPK1
quercetin	PDSS2
quercetin	DOCK4
quercetin	DOCK5
quercetin	CYP8B1
quercetin	DHRS13
quercetin	DHRS7
quercetin	DHCR7
quercetin	DHCR24
quercetin	ALDH4A1
quercetin	DENND1A
quercetin	DENR
quercetin	DTL
quercetin	DCK
quercetin	DNTTIP2
quercetin	DNASE1L1
quercetin	DEPTOR
quercetin	DEPDC1
quercetin	DEPDC1B
quercetin	DEPDC7
quercetin	DSE
quercetin	DSC2
quercetin	DSTN
quercetin	DRG2
quercetin	RASD1
quercetin	GLCE
quercetin	DGKA
quercetin	DGAT2
quercetin	SAT1
quercetin	DKK4
quercetin	DEF8
quercetin	DHFRL1
quercetin	DLD

quercetin	QDPR
quercetin	DPYSL2
quercetin	DPYSL4
quercetin	DPYD
quercetin	TFB1M
quercetin	FMO5
quercetin	CYP1B1
quercetin	CTSC
quercetin	DPP4
quercetin	MVD
quercetin	DAB2
quercetin	DCBLD2
quercetin	DIP2A
quercetin	ADAM12
quercetin	ADAM15
quercetin	ADAM9
quercetin	DLG1
quercetin	DLGAP5
quercetin	DIRC2
quercetin	ZNF850
quercetin	DMXL1
quercetin	DMXL2
quercetin	DNMT1
quercetin	DDB2
quercetin	DDIT3
quercetin	DDIT4
quercetin	DRAM1
quercetin	APOBEC3G
quercetin	RBBP8
quercetin	ERCC1
quercetin	ERCC6L
quercetin	DFFA
quercetin	DFFB
quercetin	N/A
quercetin	INO80
quercetin	MSH2
quercetin	MSH6
quercetin	POLA1
quercetin	POLD2
quercetin	POLD4
quercetin	POLE
quercetin	POLE2
quercetin	POLH
quercetin	POLQ
quercetin	REV3L
quercetin	ABCG2
quercetin	RAD54B
quercetin	RAD54L
quercetin	XPC
quercetin	ERCC5
quercetin	XRCC3
quercetin	GINS4
quercetin	CDT1
quercetin	TOP1
quercetin	ABHD14B
quercetin	TOP2A
quercetin	TOP2B
quercetin	TOP3A
quercetin	TOP3B

quercetin	TOP1MT
quercetin	SATB2
quercetin	PRKDC
quercetin	ZNRD1
quercetin	ABHD4
quercetin	POLR3B
quercetin	POLR3D
quercetin	POLR3G
quercetin	POLR1D
quercetin	DNAJA1
quercetin	DNAJB2
quercetin	DNAJB4
quercetin	DNAJC12
quercetin	DNAJC2
quercetin	ASPM
quercetin	DOK6
quercetin	DOLK
quercetin	DPM2
quercetin	DPM3
quercetin	ALG6
quercetin	ALG10
quercetin	DCDC2
quercetin	MRE11A
quercetin	DPH3
quercetin	DYDC2
quercetin	DRAP1
quercetin	DBN1
quercetin	DTWD1
quercetin	DUOX1
quercetin	DUOX2
quercetin	DSTYK
quercetin	MAP2K3
quercetin	MAP2K4
quercetin	MAP2K6
quercetin	DUSP28
quercetin	CLK1
quercetin	TTK
quercetin	DUSP1
quercetin	DUSP4
quercetin	DUSP5
quercetin	DUSP6
quercetin	TESK1
quercetin	XPO1
quercetin	DNHD1
quercetin	DYNLL1
quercetin	DYNLT3
quercetin	HERC5
quercetin	EGR2
quercetin	KIAA1586
quercetin	CCNB1IP1
quercetin	UBE3D
quercetin	FANCL
quercetin	HERC2
quercetin	MIB2
quercetin	MYLIP
quercetin	NEDD4L
quercetin	PELI3
quercetin	RBBP6
quercetin	RFWD3

quercetin	RFFL
quercetin	RNF14
quercetin	RNF146
quercetin	RNF19B
quercetin	RNF213
quercetin	RNF43
quercetin	SH3RF1
quercetin	SIAH1
quercetin	SIAH2
quercetin	SMURF1
quercetin	SMURF2
quercetin	TRIM11
quercetin	TRIM21
quercetin	TRIM22
quercetin	ZFYVE19
quercetin	TRIM4
quercetin	UBR4
quercetin	AIM1
quercetin	XIAP
quercetin	ZNRF3
quercetin	EGR1
quercetin	EGR3
quercetin	EGR4
quercetin	EML2
quercetin	ART4
quercetin	ENTPD5
quercetin	ENPP1
quercetin	ENPP2
quercetin	ENPP3
quercetin	KATNBL1
quercetin	EFCAB7
quercetin	EOGT
quercetin	EFEMP1
quercetin	EMR2
quercetin	EGLN1
quercetin	EHBP1
quercetin	ETFB
quercetin	EAF2
quercetin	ELMOD2
quercetin	EEF1A1
quercetin	GFM1
quercetin	TSFM
quercetin	TUFM
quercetin	ELOVL1
quercetin	ELOVL3
quercetin	ELOVL6
quercetin	IKBKAP
quercetin	TMEM126B
quercetin	ENDOD1
quercetin	ACAT2
quercetin	HSPA5
quercetin	ACAT1
quercetin	ERP27
quercetin	ERP29
quercetin	EVA1A
quercetin	ERGIC2
quercetin	HSP90B1
quercetin	ESAM
quercetin	ACACA

quercetin	ESM1
quercetin	LIPG
quercetin	EPAS1
quercetin	PROCR
quercetin	EDNRB
quercetin	EDN1
quercetin	NEDD9
quercetin	EDC3
quercetin	ECI2
quercetin	CLASP2
quercetin	EID2B
quercetin	SPRTN
quercetin	NGEF
quercetin	EPHA1
quercetin	EPHA10
quercetin	EPHA2
quercetin	EPHA3
quercetin	EPHA4
quercetin	EPHA5
quercetin	EPHA7
quercetin	EPHA8
quercetin	EPHB1
quercetin	EPHB2
quercetin	EPHB3
quercetin	EPHB4
quercetin	EPHB6
quercetin	EFNA1
quercetin	EFNB2
quercetin	EGFR
quercetin	EPS8
quercetin	EPS8L2
quercetin	EPS15
quercetin	ALOXE3
quercetin	EMP1
quercetin	EMP3
quercetin	SLC33A1
quercetin	EPN3
quercetin	SLC29A1
quercetin	SLC29A3
quercetin	EPO
quercetin	SELE
quercetin	ESPN
quercetin	ASAH1
quercetin	C11orf54
quercetin	HSD17B2
quercetin	ESR1
quercetin	ESR1
quercetin	ESR1
quercetin	ESR1
quercetin	ESR1
quercetin	ESR1
quercetin	ESR1
quercetin	ESR1
quercetin	ESR1
quercetin	ESR1
quercetin	ESR1
quercetin	ESR1
quercetin	ESR1
quercetin	ESR1

quercetin	ESR1
quercetin	ESR1
quercetin	ESR1
quercetin	ESR1
quercetin	ESR1
quercetin	SMPDL3A
quercetin	ESR1
quercetin	ESR1
quercetin	ESR1
quercetin	ESR1
quercetin	ESR1
quercetin	ESR1
quercetin	ESR1
quercetin	ESR1
quercetin	ESR1
quercetin	ESR1
quercetin	ESR1
quercetin	ESR1
quercetin	ESR1
quercetin	ESR1
quercetin	ESR1
quercetin	ESR1
quercetin	ESR1
quercetin	ESR1
quercetin	ESR1
quercetin	ESR1
quercetin	ESR1
quercetin	ESR1
quercetin	ESR1
quercetin	ESR1
quercetin	ESR1
quercetin	ESR1
quercetin	ESR1
quercetin	ESR1
quercetin	ESR1
quercetin	ESR1
quercetin	ESR2
quercetin	ESR2
quercetin	ESR2
quercetin	ESR2
quercetin	ESR2
quercetin	ESR2
quercetin	ESR2
quercetin	ESR2
quercetin	ESR2
quercetin	ESR1
quercetin	SULT1E1
quercetin	EI24
quercetin	ELK1
quercetin	ELK4
quercetin	ETV4
quercetin	ELF3
quercetin	ELF4
quercetin	EIF4A1
quercetin	PLCB1
quercetin	ACRC
quercetin	EIF1
quercetin	EIF2S1
quercetin	EIF2S2

quercetin	EIF3F
quercetin	EIF4E2
quercetin	EIF6
quercetin	SLC1A2
quercetin	EXOC2
quercetin	EXOC4
quercetin	EXOC6
quercetin	EXOSC8
quercetin	EXOSC9
quercetin	EXTL2
quercetin	SLC5A9
quercetin	XPOT
quercetin	SULF2
quercetin	FLAD1
quercetin	FANCB
quercetin	FANCG
quercetin	FAN1
quercetin	FDPS
quercetin	FAIM
quercetin	FEZ2
quercetin	FASTKD1
quercetin	FASTKD2
quercetin	FITM1
quercetin	ACTB
quercetin	FADS3
quercetin	FASN
quercetin	FABP1
quercetin	ALDH3A2
quercetin	FBXO10
quercetin	FBXO2
quercetin	FBXO25
quercetin	FBXO28
quercetin	FBXO3
quercetin	FBXO41
quercetin	FBXO5
quercetin	ACTA1
quercetin	FBXL2
quercetin	PDP2
quercetin	FBXL6
quercetin	ACTA2
quercetin	FCHSD2
quercetin	FLVCR2
quercetin	FRMD3
quercetin	FRMD4B
quercetin	FRMD5
quercetin	ACTB
quercetin	FARP1
quercetin	FARP2
quercetin	FERMT1
quercetin	FERMT2
quercetin	FGA
quercetin	FGB
quercetin	FGG
quercetin	FGF18
quercetin	ABLIM3
quercetin	FGF7
quercetin	FGFR1
quercetin	FGFR2
quercetin	FGFR3

quercetin	FGFR4
quercetin	FGFRL1
quercetin	ANLN
quercetin	FGFBP3
quercetin	FGL2
quercetin	FN1
quercetin	COBLL1
quercetin	FNDC3B
quercetin	FBLN2
quercetin	FIGN
quercetin	FIGNL1
quercetin	FILIP1L
quercetin	FLNA
quercetin	FLNB
quercetin	PTK2
quercetin	FLCN
quercetin	FNIP2
quercetin	FSTL3
quercetin	FOXM1
quercetin	FOXN3
quercetin	FOXP1
quercetin	FTCD
quercetin	FNBP1L
quercetin	FOSL1
quercetin	CX3CL1
quercetin	FMR1
quercetin	FZD7
quercetin	FBP1
quercetin	TIGAR
quercetin	ALDOA
quercetin	ALDOC
quercetin	FH
quercetin	FAH
quercetin	FXYD5
quercetin	GPANK1
quercetin	MICB
quercetin	CCND1
quercetin	ACTR6
quercetin	CCND2
quercetin	CCND3
quercetin	CCNE1
quercetin	CCNE2
quercetin	G2E3
quercetin	CCNB1
quercetin	CCNB2
quercetin	GABPB2
quercetin	GAL3ST1
quercetin	B3GAT3
quercetin	GALR2
quercetin	LGALS1
quercetin	LGALS2
quercetin	LGALS3
quercetin	LGALS3BP
quercetin	LGALS8
quercetin	ADD3
quercetin	SUB1
quercetin	GABRE
quercetin	ASCC3
quercetin	IFI30

quercetin	TUBGCP3
quercetin	TUBGCP6
quercetin	GJA1
quercetin	GJB1
quercetin	GAS2L3
quercetin	GSDMB
quercetin	PGC
quercetin	GCFC2
quercetin	ATF7IP
quercetin	TSTA3
quercetin	GMDS
quercetin	GSN
quercetin	GEMIN2
quercetin	GEMIN6
quercetin	AHSA1
quercetin	GSE1
quercetin	GHDC
quercetin	CCDC88A
quercetin	SERPINE2
quercetin	GLIPR1
quercetin	GLMN
quercetin	NR3C1
quercetin	GLCCI1
quercetin	PGM2L1
quercetin	G6PC
quercetin	G6PD
quercetin	GPI
quercetin	GFOD1
quercetin	GAD1
quercetin	GLUD1
quercetin	GLUD2
quercetin	GCLC
quercetin	GCLM
quercetin	GLS
quercetin	GLS2
quercetin	PDE12
quercetin	QRICH2
quercetin	QPCT
quercetin	ADNP
quercetin	GLRX2
quercetin	GPX1
quercetin	GPX2
quercetin	GSR
quercetin	GSTA1
quercetin	GSTA4
quercetin	GSTK1
quercetin	GSTM1
quercetin	GSTM2
quercetin	GSTM4
quercetin	GSTP1
quercetin	GPAM
quercetin	AGPAT9
quercetin	GPD1L
quercetin	GDPD5
quercetin	GATM
quercetin	GLDC
quercetin	GNMT
quercetin	ACAD11
quercetin	GLRA1

quercetin	PAEP
quercetin	GYS1
quercetin	AGL
quercetin	PYGB
quercetin	PYGL
quercetin	PYGM
quercetin	GSK3A
quercetin	GSK3B
quercetin	GYG2
quercetin	GLTPD2
quercetin	ARSA
quercetin	ACSF2
quercetin	MANEA
quercetin	GPHA2
quercetin	C1GALT1
quercetin	GPAA1
quercetin	GLT8D1
quercetin	NMT2
quercetin	GPC1
quercetin	GPC6
quercetin	GOLGA8A
quercetin	GBF1
quercetin	PIGN
quercetin	PGAP1
quercetin	PIGZ
quercetin	GPN1
quercetin	ACSS3
quercetin	GPR35
quercetin	GPSM2
quercetin	GCA
quercetin	LPGAT1
quercetin	CSF3R
quercetin	CSF2
quercetin	GAREM1
quercetin	ACBD6
quercetin	PLA2G12B
quercetin	PLA2G16
quercetin	GADD45A
quercetin	GADD45B
quercetin	GAS2
quercetin	GRB10
quercetin	GRB14
quercetin	DBI
quercetin	GDF11
quercetin	GDF15
quercetin	GRPEL1
quercetin	FRAT2
quercetin	HRAS
quercetin	KRAS
quercetin	NRAS
quercetin	GTPBP8
quercetin	GEM
quercetin	RRAD
quercetin	RIT2
quercetin	GDA
quercetin	GNAI1
quercetin	ACSM3
quercetin	GNG4
quercetin	ACSM5

quercetin	GNA13
quercetin	GNA15
quercetin	RACK1
quercetin	GUCA1B
quercetin	CLCN3
quercetin	CLCN5
quercetin	PDK1
quercetin	DECR1
quercetin	TSC1
quercetin	HP
quercetin	ACOT13
quercetin	HAUS1
quercetin	HAUS3
quercetin	PHF14
quercetin	HS1BP3
quercetin	HEATR1
quercetin	HEATR3
quercetin	HSPA12A
quercetin	HSPA14
quercetin	HSPA1B
quercetin	HSPA4
quercetin	HSPA4L
quercetin	HSPA2
quercetin	HSF1
quercetin	HSPH1
quercetin	HSPB1
quercetin	HSP90AA1
quercetin	HSPA2
quercetin	HLTF
quercetin	HCLS1
quercetin	HPGDS
quercetin	HHEX
quercetin	HMOX1
quercetin	ACYP1
quercetin	HBG2
quercetin	HS2ST1
quercetin	SERPIND1
quercetin	HLF
quercetin	LIPC
quercetin	MET
quercetin	HGS
quercetin	FOXA1
quercetin	FOXA3
quercetin	HNF4G
quercetin	HAMP
quercetin	ADAMTSL4
quercetin	HHLA3
quercetin	HNRNPA0
quercetin	HNRNPD
quercetin	HNRNPH1
quercetin	HNRNPH3
quercetin	HNRNPA2B1
quercetin	COQ3
quercetin	SLC51B
quercetin	HK2
quercetin	FCER1G
quercetin	HMGN3
quercetin	MRGPRX3
quercetin	HMGB1

quercetin	HMGB2
quercetin	HMGA1
quercetin	APC
quercetin	ESR1
quercetin	HNMT
quercetin	HAL
quercetin	HDC
quercetin	HINT3
quercetin	KAT2B
quercetin	ADA
quercetin	ASF1B
quercetin	SAP30L
quercetin	HIST1H1C
quercetin	H1FX
quercetin	HIST1H2AB
quercetin	HIST1H2AC
quercetin	HIST1H2BD
quercetin	RNASEL
quercetin	ZNF468
quercetin	HIST1H3A
quercetin	CENPA
quercetin	HIST1H4A
quercetin	PHF8
quercetin	SETDB2
quercetin	SETMAR
quercetin	CD74
quercetin	HLA-DMA
quercetin	HBP1
quercetin	DLX2
quercetin	DLX6
quercetin	ADORA2A
quercetin	HOXA3
quercetin	HOXA4
quercetin	HOXA5
quercetin	HOXD1
quercetin	MEIS1
quercetin	MEIS2
quercetin	MIXL1
quercetin	MSX1
quercetin	NKX3-1
quercetin	TGIF2
quercetin	HERPUD1
quercetin	PSMC3IP
quercetin	HUNK
quercetin	HCFC1R1
quercetin	CDC37
quercetin	HSP90AA1
quercetin	HMMR
quercetin	HAS1
quercetin	HAS2
quercetin	HAS3
quercetin	HYLS1
quercetin	HMGCL
quercetin	HMGCS1
quercetin	HIF1A
quercetin	HILPDA
quercetin	IER2
quercetin	IER5
quercetin	IER5L

quercetin	IGDCC3
quercetin	IGDCC4
quercetin	IGSF1
quercetin	KPNA3
quercetin	KPNA4
quercetin	IPO8
quercetin	ARL15
quercetin	RHBDF2
quercetin	PTK7
quercetin	CYP27B1
quercetin	IDO1
quercetin	MCL1
quercetin	IVNS1ABP
quercetin	INHBB
quercetin	INHBE
quercetin	CHUK
quercetin	IKBIP
quercetin	INO80B
quercetin	MALSU1
quercetin	PPA2
quercetin	ITPR1
quercetin	ITPR2
quercetin	ITPR3
quercetin	ITPRIP
quercetin	ITPRIPL1
quercetin	IP6K2
quercetin	IMPA2
quercetin	ISYNA1
quercetin	ITPKA
quercetin	INSR
quercetin	IRS1
quercetin	INSRR
quercetin	IGF1R
quercetin	IGF2BP3
quercetin	IGF2
quercetin	IGFBP1
quercetin	IGFBP2
quercetin	IGFBP3
quercetin	OAS3
quercetin	ADSSL1
quercetin	IGFBP4
quercetin	INTS8
quercetin	ITGA10
quercetin	ITGA2
quercetin	ITGA6
quercetin	ITGAV
quercetin	ITIH1
quercetin	ICAM1
quercetin	ICAM3
quercetin	ICAM4
quercetin	IFNAR1
quercetin	IFI6
quercetin	IFNG
quercetin	IFNGR2
quercetin	IRF1
quercetin	IRF2BPL
quercetin	IRF3
quercetin	IFIH1
quercetin	IFIT1

quercetin	IFIT5
quercetin	IL1A
quercetin	IL1B
quercetin	IL1R2
quercetin	IRAK1
quercetin	IRAK1BP1
quercetin	IRAK3
quercetin	IRAK2
quercetin	IL1RL1
quercetin	IL10
quercetin	IL11
quercetin	IL15
quercetin	IL17RB
quercetin	IL17A
quercetin	ADIPOQ
quercetin	IL2
quercetin	IL20RB
quercetin	IL22RA1
quercetin	IL32
quercetin	IL4
quercetin	IL4R
quercetin	IL6
quercetin	IL6ST
quercetin	IL7
quercetin	CXCL8
quercetin	ITSN1
quercetin	MMP1
quercetin	ADM
quercetin	ISX
quercetin	IFT172
quercetin	IFT80
quercetin	IQCF1
quercetin	TCF7L2
quercetin	NUBPL
quercetin	IRX3
quercetin	ISOC1
quercetin	PDK2
quercetin	IDH1
quercetin	IARS
quercetin	IDI1
quercetin	ISM2
quercetin	JAK1
quercetin	PGBD3
quercetin	JDP2
quercetin	JMY
quercetin	KLK2
quercetin	NUDT9
quercetin	SERPINA4
quercetin	KATNAL1
quercetin	KAZN
quercetin	KDELC1
quercetin	KLHDC2
quercetin	ZNF75A
quercetin	CD38
quercetin	SLC51A
quercetin	KBTBD6
quercetin	KBTBD7
quercetin	KEAP1
quercetin	KLHL14

quercetin	KLHL2
quercetin	KLHL21
quercetin	SELENOO
quercetin	EFNA4
quercetin	KLHL9
quercetin	KRT15
quercetin	KRT19
quercetin	KRT20
quercetin	KRT23
quercetin	KRT1
quercetin	ARF3
quercetin	FAM222A
quercetin	FAM135A
quercetin	PRODH2
quercetin	SLC25A30
quercetin	KLC4
quercetin	KIFAP3
quercetin	KIF11
quercetin	KIF14
quercetin	KIF15
quercetin	KIF16B
quercetin	KIF18A
quercetin	KIF20A
quercetin	KIF20B
quercetin	KIF21A
quercetin	KIF23
quercetin	KIF2A
quercetin	KIF3A
quercetin	KIFC2
quercetin	NDC80
quercetin	NUF2
quercetin	NADK2
quercetin	SPC25
quercetin	KNG1
quercetin	KANK1
quercetin	KANK4
quercetin	KRIT1
quercetin	KLF3
quercetin	KLF4
quercetin	ARL13B
quercetin	KLF5
quercetin	KLF6
quercetin	HKR1
quercetin	AADAT
quercetin	CCBL2
quercetin	LCT
quercetin	GLO1
quercetin	LAD1
quercetin	CRYL1
quercetin	LBR
quercetin	LMNB1
quercetin	LSS
quercetin	LARP6
quercetin	SLC7A5
quercetin	DBR1
quercetin	ASRGL1
quercetin	ARL4D
quercetin	YAE1D1
quercetin	LGSN

quercetin	LIM2
quercetin	LEPR
quercetin	ARL6
quercetin	LETM2
quercetin	LRG1
quercetin	LRCH1
quercetin	LRRIQ3
quercetin	FLRT3
quercetin	LGR4
quercetin	LGR5
quercetin	FAM83D
quercetin	LRRC37A3
quercetin	RRNAD1
quercetin	COX20
quercetin	LRRC8D
quercetin	C10orf11
quercetin	LRIG1
quercetin	LRIG3
quercetin	SMIM20
quercetin	LZTR1
quercetin	LNPEP
quercetin	LIF
quercetin	LIFR
quercetin	LNP1
quercetin	SERPINB1
quercetin	LENG8
quercetin	LAIR2
quercetin	LTB4R
quercetin	CYP4F2
quercetin	CYP4F3
quercetin	LPXN
quercetin	LNX2
quercetin	LMCD1
quercetin	LIMA1
quercetin	LIMK1
quercetin	LIMK2
quercetin	LIPH
quercetin	LCN15
quercetin	LSR
quercetin	PPFIBP2
quercetin	C16orf5
quercetin	LEAP2
quercetin	LONRF1
quercetin	ACSL5
quercetin	ACSL6
quercetin	FCGR2B
quercetin	LDLR
quercetin	LRP10
quercetin	LAS2
quercetin	LY96
quercetin	PBK
quercetin	KRCC1
quercetin	KDM3A
quercetin	KDM1A
quercetin	KDM1B
quercetin	LYSMD2
quercetin	LPAR6
quercetin	LYPLAL1
quercetin	TMEM86B

quercetin	LIPA
quercetin	GAA
quercetin	AGMAT
quercetin	LAPTM5
quercetin	LYZ
quercetin	DCPS
quercetin	CSF1R
quercetin	MIF
quercetin	MPP5
quercetin	MPP6
quercetin	MPP7
quercetin	MFSD10
quercetin	MFSD8
quercetin	MR1
quercetin	MVP
quercetin	MFHAS1
quercetin	AKAP12
quercetin	MGAM
quercetin	C17orf48
quercetin	GMPPB
quercetin	MAN1A1
quercetin	MAN1A2
quercetin	MANSC1
quercetin	MKNK2
quercetin	MBIP
quercetin	MAP7D1
quercetin	KIT
quercetin	MELK
quercetin	MMP7
quercetin	AKIRIN1
quercetin	MMP9
quercetin	AKIRIN2
quercetin	MXD1
quercetin	DTD2
quercetin	MXI1
quercetin	TMEM67
quercetin	MED10
quercetin	MED21
quercetin	MED26
quercetin	MED30
quercetin	MED7
quercetin	MNS1
quercetin	MIA2
quercetin	GPT2
quercetin	MAGED1
quercetin	MLPH
quercetin	CD46
quercetin	AOC3
quercetin	PAQR8
quercetin	PAQR5
quercetin	FAM174A
quercetin	MAGI2
quercetin	MAGI3
quercetin	PITPNM1
quercetin	PKMYT1
quercetin	TMEM50B
quercetin	MBLAC2
quercetin	OMA1
quercetin	TIMP1

quercetin	TIMP2
quercetin	PDK3
quercetin	PSMD2
quercetin	STEAP1
quercetin	STEAP2
quercetin	MT1G
quercetin	MT1M
quercetin	MT2A
quercetin	MTSS1
quercetin	ALB
quercetin	MTA1
quercetin	METRNL
quercetin	AKR1A1
quercetin	MTR
quercetin	MARS2
quercetin	MBD4
quercetin	MBD6
quercetin	TET1
quercetin	ADH4
quercetin	AUH
quercetin	ALDH6A1
quercetin	MSMO1
quercetin	RFPL3S
quercetin	MRI1
quercetin	CHCHD10
quercetin	METTL5
quercetin	METTL7A
quercetin	METTL7B
quercetin	MICALL1
quercetin	MICALL2
quercetin	ADH5
quercetin	MITF
quercetin	DGCR8
quercetin	PSMD3
quercetin	MGST2
quercetin	MTTP
quercetin	MAP1B
quercetin	MAP1LC3B
quercetin	MTUS1
quercetin	MDK
quercetin	MIDN
quercetin	HMHA1
quercetin	MIPOL1
quercetin	MITD1
quercetin	SPATA18
quercetin	SLC25A21
quercetin	MARC1
quercetin	SLC25A20
quercetin	ENOSF1
quercetin	MTFR1
quercetin	TIMM8A
quercetin	TOMM22
quercetin	XRCC6BP1
quercetin	IMMP1L
quercetin	ALDH1B1
quercetin	IMMT
quercetin	RHOT1
quercetin	TRMU
quercetin	UCP2

quercetin	MAPK1
quercetin	ALDH2
quercetin	MAPK11
quercetin	MAPK12
quercetin	MAPK13
quercetin	MAPK14
quercetin	MAPK15
quercetin	MAPK3
quercetin	MAPK4
quercetin	MAPK6
quercetin	MAPK7
quercetin	MAPK8
quercetin	MAP3K10
quercetin	MAP3K11
quercetin	MAP3K12
quercetin	MAP3K13
quercetin	MAP3K4
quercetin	AOX1
quercetin	MAP3K7
quercetin	MAP3K8
quercetin	MAP3K9
quercetin	MAP4K1
quercetin	MAP4K4
quercetin	BUB3
quercetin	BUB1
quercetin	BUB1B
quercetin	MAD1L1
quercetin	MAD2L1
quercetin	MLKL
quercetin	MOB1B
quercetin	AKR1B10
quercetin	MOCOS
quercetin	MOCS2
quercetin	ABHD6
quercetin	SLC16A1
quercetin	AKR1B15
quercetin	SLC16A7
quercetin	SLC16A3
quercetin	AKR1C1
quercetin	SLC16A4
quercetin	SLC16A6
quercetin	MORC4
quercetin	MORN2
quercetin	KLHL5
quercetin	SMAD5
quercetin	SMAD7
quercetin	AKR1C2
quercetin	CDC25C
quercetin	AKR1C3
quercetin	DCP1B
quercetin	MTERFD1
quercetin	MCOLN1
quercetin	AKR1C4
quercetin	MALT1
quercetin	ABCG2
quercetin	ABCB1
quercetin	ABCB4
quercetin	ABCC1
quercetin	ABCC4

quercetin	ABCC6
quercetin	MEGF6
quercetin	PSMD5
quercetin	GALM
quercetin	MEGF8
quercetin	MPDZ
quercetin	AKR1B1
quercetin	MUSK
quercetin	MBNL2
quercetin	MBNL3
quercetin	CYP1B1
quercetin	MSH5
quercetin	MSANTD1
quercetin	MSANTD3
quercetin	MYBL1
quercetin	MYC
quercetin	MPZ
quercetin	MPO
quercetin	MYOF
quercetin	MYL5
quercetin	MYLK
quercetin	MYL12A
quercetin	MYL9
quercetin	DMPK
quercetin	MTMR4
quercetin	SLC9A3R1
quercetin	RIMKLA
quercetin	NAALAD2
quercetin	GNPTAB
quercetin	NAGS
quercetin	GCNT2
quercetin	NAT14
quercetin	NAT6
quercetin	NAT9
quercetin	NAPEPLD
quercetin	NQO1
quercetin	NQO2
quercetin	RETSAT
quercetin	ME2
quercetin	SIRT1
quercetin	SIRT2
quercetin	SIRT3
quercetin	SIRT4
quercetin	SIRT6
quercetin	SIRT7
quercetin	NDUFA3
quercetin	NDUFA4
quercetin	NDUFS1
quercetin	NOX1
quercetin	NOX3
quercetin	FUT9
quercetin	NOX4
quercetin	NOX5
quercetin	POR
quercetin	NAA15
quercetin	NELF
quercetin	SLC11A2
quercetin	NCKAP1
quercetin	NCKIPSD

quercetin	LBH
quercetin	NEB
quercetin	NDFIP2
quercetin	N4BP2L1
quercetin	N4BP2L2
quercetin	NEDD8
quercetin	UBA3
quercetin	FUT11
quercetin	RDBP
quercetin	FUT8
quercetin	NTN4
quercetin	PPP1R9A
quercetin	NBEA
quercetin	SCG5
quercetin	NET1
quercetin	NEFM
quercetin	NMB
quercetin	NAV2
quercetin	CHRNA10
quercetin	NREP
quercetin	NPY1R
quercetin	NETO2
quercetin	SERPINI1
quercetin	ORM1
quercetin	NCF1
quercetin	NCF2
quercetin	NEXN
quercetin	ORM2
quercetin	NFKBIA
quercetin	NKIRAS1
quercetin	NKIRAS2
quercetin	FPR2
quercetin	OGG1
quercetin	NBN
quercetin	NAMPT
quercetin	A1BG
quercetin	NPC1L1
quercetin	NIF3L1
quercetin	NIN
quercetin	NINJ2
quercetin	NOS1
quercetin	NOS3
quercetin	NOS2
quercetin	SETD6
quercetin	NMI
quercetin	TYK2
quercetin	DFNA5
quercetin	NMES1
quercetin	PDK4
quercetin	NLE1
quercetin	NUAK1
quercetin	NFE2L2
quercetin	NFE2L3
quercetin	NFIL3
quercetin	NFKB2
quercetin	SERPINF2
quercetin	NUP85
quercetin	ADAL
quercetin	NUP210

quercetin	NUPR1
quercetin	MDM1
quercetin	MPC1
quercetin	NCOA1
quercetin	NCOA2
quercetin	AHSG
quercetin	NR0B2
quercetin	NR1I2
quercetin	NR1I3
quercetin	NR1I2
quercetin	NR4A1
quercetin	NSRP1
quercetin	NUSAP1
quercetin	TIA1
quercetin	NUP35
quercetin	CDC27
quercetin	NUP62CL
quercetin	ZNF211
quercetin	NME7
quercetin	NME4
quercetin	NAP1L5
quercetin	BPTF
quercetin	AASS
quercetin	NACC2
quercetin	NUDCD2
quercetin	MACROD1
quercetin	ODAM
quercetin	OLFML1
quercetin	OLFML3
quercetin	PPP1R21
quercetin	CEP89
quercetin	SYNE4
quercetin	OMG
quercetin	NIT2
quercetin	SEPSECS
quercetin	OGFRL1
quercetin	ACTR1A
quercetin	KBTBD3
quercetin	KBTBD4
quercetin	ORC1
quercetin	ORC2
quercetin	ORC6
quercetin	ORMDL2
quercetin	ODC1
quercetin	NR1I2
quercetin	BGLAP
quercetin	OSTF1
quercetin	OSTM1
quercetin	SPP1
quercetin	OAF
quercetin	AK4
quercetin	OXR1
quercetin	ENO1
quercetin	OSGIN2
quercetin	OXNAD1
quercetin	OSBPL10
quercetin	OSBPL11
quercetin	OSBPL3
quercetin	OSBPL8

quercetin	GLA
quercetin	OXTR
quercetin	P2RY2
quercetin	PERP
quercetin	PAN2
quercetin	SIN3A
quercetin	PAX8
quercetin	PALLD
quercetin	PALMD
quercetin	ZDHHC2
quercetin	ZDHHC21
quercetin	PDX1
quercetin	SPINK1
quercetin	VNN1
quercetin	PANK1
quercetin	PANK2
quercetin	NR1I2
quercetin	CDC73
quercetin	PNMA1
quercetin	PTHLH
quercetin	WIBG
quercetin	PASK
quercetin	PNPLA3
quercetin	PSIP1
quercetin	MAN2C1
quercetin	PDLIM7
quercetin	GIPC1
quercetin	GIPC2
quercetin	PCNXL3
quercetin	NGLY1
quercetin	PPIA
quercetin	PPID
quercetin	FKBP10
quercetin	FKBP8
quercetin	PIN1
quercetin	PPIL4
quercetin	PPWD1
quercetin	PWP1
quercetin	PWP2
quercetin	PMP22
quercetin	NAGLU
quercetin	PRDX2
quercetin	PRDX4
quercetin	PRDX5
quercetin	ACOX2
quercetin	ACOX3
quercetin	EHHADH
quercetin	PEX3
quercetin	PEX13
quercetin	HSD17B4
quercetin	PIPOX
quercetin	PECR
quercetin	PEX6
quercetin	PEX2
quercetin	ALPK2
quercetin	PPARA
quercetin	PPARD
quercetin	PPARG
quercetin	PPARGC1A

quercetin	ABCB1
quercetin	PSD3
quercetin	PSD4
quercetin	PHF10
quercetin	PAH
quercetin	PMAIP1
quercetin	PHACTR2
quercetin	MROH1
quercetin	TXLNA
quercetin	CDS1
quercetin	PEBP1
quercetin	PTPMT1
quercetin	PTEN
quercetin	PIK3C3
quercetin	PIK3CA
quercetin	PIK3CB
quercetin	PIK3CD
quercetin	PIK3CG
quercetin	PIK3C2A
quercetin	PIK3C2B
quercetin	PIK3C2G
quercetin	PIP5K1B
quercetin	ALMS1
quercetin	PCK1
quercetin	PGM1
quercetin	PGM2
quercetin	PGK1
quercetin	PGAM1
quercetin	PIK3AP1
quercetin	PIK3CG
quercetin	PLA2G1B
quercetin	PLA2G2A
quercetin	PLD1
quercetin	PLD3
quercetin	PLSCR1
quercetin	PLSCR4
quercetin	PLTP
quercetin	PPCDC
quercetin	PHKA2
quercetin	PHKB
quercetin	AHR
quercetin	PSAT1
quercetin	PSPH
quercetin	PPP1R18
quercetin	PHYHD1
quercetin	PHYHIPL
quercetin	PGBD1
quercetin	SERPINF1
quercetin	PIR
quercetin	PITHD1
quercetin	PITX2
quercetin	PLAC8L1
quercetin	PKP2
quercetin	PKP4
quercetin	SERPINE1
quercetin	SERPINB2
quercetin	PLS1
quercetin	PEAR1
quercetin	PAFAH1B1

quercetin	PDGFC
quercetin	PDGFRA
quercetin	PDGFRB
quercetin	PDGFRL
quercetin	PLEKHA2
quercetin	PLEKHA6
quercetin	ESR1
quercetin	PLEKHH1
quercetin	PLEKHM1
quercetin	PLEKHO1
quercetin	PHLDA1
quercetin	PHLDA2
quercetin	PHLDA3
quercetin	PHLDB2
quercetin	DKFZp686L2375
quercetin	PLXNA3
quercetin	PLXNB2
quercetin	PLXNC1
quercetin	PVRL4
quercetin	PARP1
quercetin	PARP12
quercetin	PABPC1L
apigenin	POU5F1
apigenin	MAOA
apigenin	MAOB
apigenin	EGF
apigenin	POMC
apigenin	PTGS2
apigenin	PSCA
apigenin	PSME3
apigenin	RUNX1T1
apigenin	FOS
apigenin	SRC
apigenin	TP53I3
apigenin	AKT1
apigenin	FLT3
apigenin	RB1
apigenin	RPS6KB1
apigenin	SLC5A5
apigenin	FXYD2
apigenin	SLC2A1
apigenin	SLC2A4
apigenin	BAX
apigenin	BCL2
apigenin	CXCL12
apigenin	C11orf80
apigenin	VEGFA
apigenin	GYPA
apigenin	SOCS3
apigenin	TNKS2
apigenin	JUN
apigenin	RELA
apigenin	TAGLN
apigenin	TRPM2
apigenin	TNF
apigenin	TNFRSF10B
apigenin	FAS
apigenin	TP63
apigenin	TP73

apigenin	AXL
apigenin	PTPN11
apigenin	UGT1A1
apigenin	UGT1A6
apigenin	ABCG2
apigenin	PLAU
apigenin	UTRN
apigenin	VEGFA
apigenin	VDR
apigenin	WISP2
apigenin	XDH
apigenin	BAD
apigenin	BCL2L1
apigenin	B3GALT5
apigenin	GUSB
apigenin	MANBA
apigenin	CSNK2A1
apigenin	CFLAR
apigenin	CASP3
apigenin	CASP9
apigenin	CAT
apigenin	CD40LG
apigenin	TP53
apigenin	CXCR4
apigenin	CDK1
apigenin	CDK4
apigenin	CDKN2C
apigenin	CDK6
apigenin	CDKN1A
apigenin	CDKN2A
apigenin	CFTR
apigenin	CYCS
apigenin	CYP11B1
apigenin	CYP19A1
apigenin	CYP1A1
apigenin	CYP1A2
apigenin	CYP3A4
apigenin	TOP1
apigenin	TOP2A
apigenin	TOP2B
apigenin	TOP3A
apigenin	TOP3B
apigenin	TOP1MT
apigenin	ALG5
apigenin	HERC5
apigenin	MDM2
apigenin	XIAP
apigenin	ELAVL1
apigenin	ACHE
apigenin	ACACA
apigenin	HSD17B1
apigenin	ESR1
apigenin	ESR2
apigenin	EEF1E1
apigenin	EIF6
apigenin	PTK2
apigenin	FOXO1
apigenin	FBP1
apigenin	CCND1

apigenin	CCND2
apigenin	CCNB1
apigenin	GMNN
apigenin	AHSA1
apigenin	G6PC
apigenin	GAD1
apigenin	GCLC
apigenin	GSK3B
apigenin	HMOX1
apigenin	MS4A2
apigenin	APC
apigenin	SPAM1
apigenin	HIF1A
apigenin	MCL1
apigenin	IKBKB
apigenin	INS
apigenin	INSR
apigenin	IGF1R
apigenin	ICAM1
apigenin	IFNG
apigenin	IL13
apigenin	IL2
apigenin	IL4
apigenin	MMP1
apigenin	ALPI
apigenin	CD38
apigenin	FCER2
apigenin	LTA
apigenin	KARS
apigenin	MMP9
apigenin	PSMD3
apigenin	MAPK1
apigenin	MAPK3
apigenin	AKR1C3
apigenin	ABCB1
apigenin	ABCC1
apigenin	AAGAB
apigenin	NOX4
apigenin	IKBKG
apigenin	NFKBIA
apigenin	NR1I2
apigenin	ODC1
apigenin	SERPINE1
apigenin	PARP1
Stigmasterol	SLCO1B1
Stigmasterol	ABCA1
Stigmasterol	ABCG5
Stigmasterol	ABCG8
Stigmasterol	HMGCR
kaempferol	POU5F1
kaempferol	PGR
kaempferol	PTGES
kaempferol	PTGS2
kaempferol	PSCA
kaempferol	GREB1
kaempferol	SRC
kaempferol	TP53I3
kaempferol	AKT1
kaempferol	RB1

kaempferol	RPS6KA3
kaempferol	UGT8
kaempferol	SLPI
kaempferol	PPP3CA
kaempferol	STAT1
kaempferol	STAT3
kaempferol	SLC2A1
kaempferol	SLC2A4
kaempferol	BAX
kaempferol	BCL2
kaempferol	ALOX12
kaempferol	ALOX15
kaempferol	ALOX5
kaempferol	SOD1
kaempferol	AKR1E2
kaempferol	AHR
kaempferol	TFAM
kaempferol	JUN
kaempferol	RELA
kaempferol	TFF1
kaempferol	TYW1
kaempferol	TNF
kaempferol	TNFRSF11B
kaempferol	TP63
kaempferol	TP73
kaempferol	DIO1
kaempferol	UGT3A1
kaempferol	UGT3A1
kaempferol	UGT1A1
kaempferol	UGT1A10
kaempferol	UGT1A3
kaempferol	UGT1A4
kaempferol	UGT1A5
kaempferol	UGT1A6
kaempferol	UGT1A7
kaempferol	UGT1A8
kaempferol	UGT1A9
kaempferol	UGT2A1
kaempferol	UGT2A3
kaempferol	UGT2B10
kaempferol	UGT2B11
kaempferol	UGT2B15
kaempferol	UGT2B17
kaempferol	UGT2B28
kaempferol	UGT2B4
kaempferol	UGT2B7
kaempferol	UGT3A1
kaempferol	UGT3A2
kaempferol	ABCG2
kaempferol	UTRN
kaempferol	VCAM1
kaempferol	WISP2
kaempferol	XDH
kaempferol	B3GALT5
kaempferol	CASP3
kaempferol	CCL2
kaempferol	SFN
kaempferol	TP53
kaempferol	CRP

kaempferol	CDK1
kaempferol	CDK2
kaempferol	CDKN2C
kaempferol	CDK6
kaempferol	CDKN1A
kaempferol	CYP1A1
kaempferol	CYP1A2
kaempferol	CYP1B1
kaempferol	CYP2B6
kaempferol	CYP3A4
kaempferol	MMP2
kaempferol	SELE
kaempferol	HSD17B1
kaempferol	HSD17B2
kaempferol	ESR1
kaempferol	ESR2
kaempferol	FBP1
kaempferol	CCNB1
kaempferol	AHSA1
kaempferol	GCLC
kaempferol	GSTA1
kaempferol	GSTM1
kaempferol	GSTM2
kaempferol	GSTP1
kaempferol	GADD45B
kaempferol	DBI
kaempferol	HMOX1
kaempferol	HAS2
kaempferol	IKBKB
kaempferol	INSR
kaempferol	IGF1R
kaempferol	IGF2
kaempferol	ITGA2
kaempferol	ICAM1
kaempferol	IL2
kaempferol	BBC3
kaempferol	MMP1
kaempferol	MT2A
kaempferol	AKR1A1
kaempferol	MTRR
kaempferol	PSMD3
kaempferol	UCP3
kaempferol	MAPK8
kaempferol	AKR1B10
kaempferol	AKR1C3
kaempferol	ABCC1
kaempferol	AKR1B1
kaempferol	NOX4
kaempferol	POR
kaempferol	NDOR1
kaempferol	NOS1
kaempferol	NOS3
kaempferol	NOS2
kaempferol	NFE2L2
kaempferol	NR1I2
kaempferol	NR1I3
kaempferol	N/A
kaempferol	PPARG
kaempferol	PPARGC1A

Trifolin	ABCG2
Trifolin	ABCG2
Trifolin	ABCG2
Trifolin	ABCG2
Trifolin	ABCG2
Trifolin	ALDH2
Trifolin	ALDH2
Trifolin	ALDH2
Trifolin	ALDH2
Trifolin	ABCG2
PEL	ADH6
PEL	TAAR1
PEL	ADH1B
ursolic acid	PTGER3
ursolic acid	PTGS1
ursolic acid	PTGS2
ursolic acid	PRKCG
ursolic acid	FOS
ursolic acid	RPS6KB1
ursolic acid	ERN1
ursolic acid	STAT3
ursolic acid	BAX
ursolic acid	BCL2
ursolic acid	MMP3
ursolic acid	MMP10
ursolic acid	SCLY
ursolic acid	BAK1
ursolic acid	JUN
ursolic acid	RELA
ursolic acid	TGFB1
ursolic acid	TNF
ursolic acid	FASLG
ursolic acid	PTPN1
ursolic acid	PTPN2
ursolic acid	PTPN6
ursolic acid	PLAU
ursolic acid	VEGFA
ursolic acid	BIRC5
ursolic acid	FGF2
ursolic acid	BCL2L1
ursolic acid	CASP1
ursolic acid	CASP3
ursolic acid	CASP8
ursolic acid	CASP9
ursolic acid	CTSB
ursolic acid	TP53
ursolic acid	MAPK8IP2
ursolic acid	ATF2
ursolic acid	CREB1
ursolic acid	CDK4
ursolic acid	CDK6
ursolic acid	CDKN1A
ursolic acid	CDKN1B
ursolic acid	CYP2C19
ursolic acid	MMP2
ursolic acid	DDIT3
ursolic acid	TOP1
ursolic acid	TOP2A
ursolic acid	DUOX2

ursolic acid	HERC5
ursolic acid	ENPP7
ursolic acid	HSPA5
ursolic acid	SELE
ursolic acid	EIF4EBP1
ursolic acid	FASN
ursolic acid	CCND1
ursolic acid	CCND2
ursolic acid	CCND3
ursolic acid	CCNE1
ursolic acid	CSF2
ursolic acid	MCL1
ursolic acid	ICAM1
ursolic acid	IL1B
ursolic acid	IL6
ursolic acid	MMP1
ursolic acid	LITAF
ursolic acid	MMP9
ursolic acid	MAPK8
ursolic acid	AKR1B10
ursolic acid	GAP43
ursolic acid	NFKBIA
ursolic acid	NOS3
ursolic acid	RORC
ursolic acid	GPBAR1
ursolic acid	INPPL1
ursolic acid	PECAM1
caffeic acid	ABHD12B
caffeic acid	ABHD13
caffeic acid	ABHD17A
caffeic acid	ABHD17B
caffeic acid	ABHD17C
caffeic acid	PRKCB
caffeic acid	SPNS1
caffeic acid	SELP
caffeic acid	RAC1
caffeic acid	SLC17A3
caffeic acid	SLC2A1
caffeic acid	SLC2A14
caffeic acid	SLC2A2
caffeic acid	SLC2A3
caffeic acid	SLC2A4
caffeic acid	SLC2A5
caffeic acid	SLC2A7
caffeic acid	SLC2A9
caffeic acid	SLC22A1
caffeic acid	SLC22A10
caffeic acid	SLC22A11
caffeic acid	SLC22A12
caffeic acid	SLC22A2
caffeic acid	SLC22A23
caffeic acid	SLC22A25
caffeic acid	SLC22A6
caffeic acid	SLC22A7
caffeic acid	SLC22A8
caffeic acid	SLC22A9
caffeic acid	ALOX15
caffeic acid	SLC16A12
caffeic acid	ALOX5

caffeic acid	N/A
caffeic acid	SV2B
caffeic acid	SV2C
caffeic acid	TNF
caffeic acid	TYR
caffeic acid	BTK
caffeic acid	SLC16A11
caffeic acid	SLC2A11
caffeic acid	SPNS2
caffeic acid	SLC22A24
caffeic acid	BAAT
caffeic acid	CYP1A1
caffeic acid	PGD
caffeic acid	MMP2
caffeic acid	MIF
caffeic acid	SLC33A1
caffeic acid	GFAP
caffeic acid	G6PD
caffeic acid	ACOT1
caffeic acid	ACOT2
caffeic acid	ACOT4
caffeic acid	HAL
caffeic acid	IGF2
caffeic acid	MMP1
caffeic acid	MIF
caffeic acid	MFSD1
caffeic acid	MFSD3
caffeic acid	MMP9
caffeic acid	MAPK1
caffeic acid	MAPK8
caffeic acid	ABHD12
caffeic acid	SLC16A1
caffeic acid	SLC16A10
caffeic acid	SLC16A8
caffeic acid	SLC16A5
caffeic acid	SLC16A6
caffeic acid	SLC16A9
caffeic acid	MPO
caffeic acid	NOS3
caffeic acid	NDP
caffeic acid	ACOT6
caffeic acid	SPNS3
protocatechuic acid	PRKCA
protocatechuic acid	PRKCB
protocatechuic acid	PRKCG
protocatechuic acid	PRKCZ
protocatechuic acid	NR5A1
protocatechuic acid	CA14
protocatechuic acid	CA2
protocatechuic acid	COMT
protocatechuic acid	DHODH
protocatechuic acid	DPYD
protocatechuic acid	MGAM
protocatechuic acid	MPO
protocatechuic acid	ENO1
protocatechualdehyde	ODC1
hexanoic acid	PRSS33
hexanoic acid	PRSS36
hexanoic acid	TISP43

hexanoic acid	GPR132
hexanoic acid	GNRH1
hexanoic acid	GNRH2
hexanoic acid	PROKR1
hexanoic acid	PROKR2
hexanoic acid	PROK1
hexanoic acid	PROK2
hexanoic acid	PMCH
hexanoic acid	MLN
hexanoic acid	PRSS37
hexanoic acid	PTGER1
hexanoic acid	PTGFR
hexanoic acid	PRSS8
hexanoic acid	KLK3
hexanoic acid	TAC1
hexanoic acid	TMPRSS12
hexanoic acid	ABHD12B
hexanoic acid	ABHD13
hexanoic acid	ABHD17A
hexanoic acid	ABHD17B
hexanoic acid	ABHD17C
hexanoic acid	APP
hexanoic acid	KLK12
hexanoic acid	F2R
hexanoic acid	F2RL1
hexanoic acid	F2RL2
hexanoic acid	F2RL3
hexanoic acid	F2
hexanoic acid	TRH
hexanoic acid	AGT
hexanoic acid	GPR65
hexanoic acid	P2RY10
hexanoic acid	QRFPR
hexanoic acid	ANXA1
hexanoic acid	PRSS27
hexanoic acid	PRSS38
hexanoic acid	PRSS53
hexanoic acid	HPN
hexanoic acid	SAA1
hexanoic acid	SH2D4A
hexanoic acid	PRSS48
hexanoic acid	LPA
hexanoic acid	PRSS57
hexanoic acid	TMPRSS11A
hexanoic acid	TRIM66
hexanoic acid	HPR
hexanoic acid	MTRNR2L12
hexanoic acid	TACR2
hexanoic acid	TACR1
hexanoic acid	ST14
hexanoic acid	TAC3
hexanoic acid	PRSS21
hexanoic acid	TBXA2R
hexanoic acid	TRHR
hexanoic acid	PLAT
hexanoic acid	TMPRSS11D
hexanoic acid	TMPRSS11E
hexanoic acid	TMPRSS2
hexanoic acid	TMPRSS3

hexanoic acid	TMPRSS4
hexanoic acid	TMPRSS5
hexanoic acid	TMPRSS6
hexanoic acid	TMPRSS9
hexanoic acid	PRSS1
hexanoic acid	PRSS3
hexanoic acid	TPSAB1
hexanoic acid	TPSD1
hexanoic acid	TPSG1
hexanoic acid	AGTR1
hexanoic acid	N/A
hexanoic acid	GPR17
hexanoic acid	PLAU
hexanoic acid	UTS2
hexanoic acid	UTS2R
hexanoic acid	UTS2B
hexanoic acid	LTB4R2
hexanoic acid	AVPR1A
hexanoic acid	AVP
hexanoic acid	PROC
hexanoic acid	PROZ
hexanoic acid	TMPRSS11B
hexanoic acid	CORIN
hexanoic acid	PRSS58
hexanoic acid	ZNF552
hexanoic acid	TMPRSS11F
hexanoic acid	AZU1
hexanoic acid	PRSS46
hexanoic acid	KLK9
hexanoic acid	BDKRB1
hexanoic acid	CTNNBL1
hexanoic acid	BAAT
hexanoic acid	PRSS22
hexanoic acid	PRSS50
hexanoic acid	OXSM
hexanoic acid	CTSG
hexanoic acid	CFB
hexanoic acid	XCR1
hexanoic acid	CCK
hexanoic acid	CCKAR
hexanoic acid	CMA1
hexanoic acid	CTRC
hexanoic acid	CELA1
hexanoic acid	CELA2A
hexanoic acid	CELA2B
hexanoic acid	CELA3A
hexanoic acid	CELA3B
hexanoic acid	CTRL
hexanoic acid	CTRB1
hexanoic acid	CTRB2
hexanoic acid	HTR2A
hexanoic acid	HTR2B
hexanoic acid	F9
hexanoic acid	HTR2C
hexanoic acid	F7
hexanoic acid	F10
hexanoic acid	F11
hexanoic acid	F12
hexanoic acid	C1RL

hexanoic acid	C1S
hexanoic acid	C2
hexanoic acid	CFB
hexanoic acid	CFD
hexanoic acid	CFI
hexanoic acid	CYSLTR1
hexanoic acid	CYSLTR2
hexanoic acid	XCL2
hexanoic acid	EDNRB
hexanoic acid	EDN1
hexanoic acid	EDNRA
hexanoic acid	EDN2
hexanoic acid	EDN3
hexanoic acid	TMPRSS15
hexanoic acid	ACR
hexanoic acid	NPVF
hexanoic acid	FFAR1
hexanoic acid	FFAR2
hexanoic acid	FFAR3
hexanoic acid	GAST
hexanoic acid	CCKBR
hexanoic acid	GRP
hexanoic acid	GRPR
hexanoic acid	GCG
hexanoic acid	GCGR
hexanoic acid	GNRHR
hexanoic acid	GPR4
hexanoic acid	GPRC6A
hexanoic acid	GZMA
hexanoic acid	GZMB
hexanoic acid	GZMH
hexanoic acid	GZMK
hexanoic acid	GZMM
hexanoic acid	GRB2
hexanoic acid	GHSR
hexanoic acid	GNG2
hexanoic acid	GNAQ
hexanoic acid	GNA11
hexanoic acid	GNA14
hexanoic acid	GNA15
hexanoic acid	ACOT1
hexanoic acid	HP
hexanoic acid	ACOT2
hexanoic acid	ACOT4
hexanoic acid	HGF
hexanoic acid	HGFAC
hexanoic acid	PRSS42
hexanoic acid	HRH1
hexanoic acid	PRSS45
hexanoic acid	HABP2
hexanoic acid	ISYNA1
hexanoic acid	KLK1
hexanoic acid	KLK10
hexanoic acid	KLK11
hexanoic acid	KLK13
hexanoic acid	KLK15
hexanoic acid	KLK2
hexanoic acid	KLK5
hexanoic acid	KLK6

hexanoic acid	KLK7
hexanoic acid	KLK8
hexanoic acid	TMPRSS7
hexanoic acid	KNG1
hexanoic acid	KISS1R
hexanoic acid	KLK14
hexanoic acid	LTB4R
hexanoic acid	XCL1
hexanoic acid	LPAR1
hexanoic acid	LPAR2
hexanoic acid	LPAR3
hexanoic acid	LPAR5
hexanoic acid	LPAR6
hexanoic acid	MST1
hexanoic acid	MASP1
hexanoic acid	MASP2
hexanoic acid	MCHR1
hexanoic acid	MCHR2
hexanoic acid	OPN4
hexanoic acid	GRM1
hexanoic acid	GRM5
hexanoic acid	KISS1
hexanoic acid	CASR
hexanoic acid	ABHD12
hexanoic acid	MLNR
hexanoic acid	AKR1B1
hexanoic acid	CHRM1
hexanoic acid	CHRM3
hexanoic acid	CHRM5
hexanoic acid	PRTN3
hexanoic acid	OVCH1
hexanoic acid	NMB
hexanoic acid	NMBR
hexanoic acid	TACR3
hexanoic acid	NMS
hexanoic acid	NMU
hexanoic acid	NMUR1
hexanoic acid	NMUR2
hexanoic acid	PRSS55
hexanoic acid	NPFFR1
hexanoic acid	NPFFR2
hexanoic acid	ADRA1A
hexanoic acid	NPS
hexanoic acid	NPSR1
hexanoic acid	NTSR1
hexanoic acid	NTSR2
hexanoic acid	NTS
hexanoic acid	PRSS12
hexanoic acid	ELANE
hexanoic acid	FPR2
hexanoic acid	ADRA1B
hexanoic acid	ADRA1D
hexanoic acid	ACOT6
hexanoic acid	QRFP
hexanoic acid	HCRT
hexanoic acid	HCRTR1
hexanoic acid	HCRTR2
hexanoic acid	GPR68
hexanoic acid	OXTR

hexanoic acid	OXT
hexanoic acid	P2RY1
hexanoic acid	P2RY2
hexanoic acid	P2RY6
hexanoic acid	KLKB1
hexanoic acid	PLG
hexanoic acid	PTAFR
palmitic acid	PPARA
palmitic acid	GPR101
palmitic acid	GPR132
palmitic acid	GPR139
palmitic acid	GPR148
palmitic acid	GPR151
palmitic acid	GPR176
palmitic acid	GPR19
palmitic acid	GPR21
palmitic acid	GPR27
palmitic acid	GPR37
palmitic acid	GPR45
palmitic acid	GPR52
palmitic acid	GPR61
palmitic acid	GPR62
palmitic acid	GPR63
palmitic acid	GPR83
palmitic acid	GPR85
palmitic acid	GPR88
palmitic acid	PAEP
palmitic acid	GNRH1
palmitic acid	GNRH2
palmitic acid	PROKR1
palmitic acid	PROKR2
palmitic acid	PROK1
palmitic acid	PROK2
palmitic acid	PRLHR
palmitic acid	PMCH
palmitic acid	MLN
palmitic acid	PTGIR
palmitic acid	PTGDR
palmitic acid	PTGES
palmitic acid	PTGER1
palmitic acid	PTGER2
palmitic acid	PTGER3
palmitic acid	PTGER4
palmitic acid	PTGFR
palmitic acid	PTGS1
palmitic acid	PTGS2
palmitic acid	PTGDS
palmitic acid	TAC1
palmitic acid	APP
palmitic acid	GPR22
palmitic acid	PRKACA
palmitic acid	PKDCC
palmitic acid	PRKACA
palmitic acid	AR
palmitic acid	TEK
palmitic acid	F2R
palmitic acid	F2RL1
palmitic acid	F2RL2
palmitic acid	F2RL3

palmitic acid	ANGPTL4
palmitic acid	TESK2
palmitic acid	ICMT
palmitic acid	PTK6
palmitic acid	PRG3
palmitic acid	TRH
palmitic acid	MAP3K21
palmitic acid	MOS
palmitic acid	AGT
palmitic acid	RET
palmitic acid	SRC
palmitic acid	GPR65
palmitic acid	SFTPA1
palmitic acid	SFTPA2
palmitic acid	GUSBP1
palmitic acid	HCK
palmitic acid	HKDC1
palmitic acid	ASAH2B
palmitic acid	P2RY10
palmitic acid	QRFPR
palmitic acid	RBP1
palmitic acid	PDHA1
palmitic acid	AKT1
palmitic acid	RGL1
palmitic acid	RAB1A
palmitic acid	RAB1B
palmitic acid	RAB33A
palmitic acid	RAB33B
palmitic acid	RAB43
palmitic acid	ANKRD1
palmitic acid	RIPK1
palmitic acid	RIPK2
palmitic acid	RIPK3
palmitic acid	RIPK4
palmitic acid	FLT3
palmitic acid	RCVRN
palmitic acid	REG3A
palmitic acid	REG4
palmitic acid	RXFP3
palmitic acid	RXFP4
palmitic acid	REN
palmitic acid	RXRA
palmitic acid	RPE65
palmitic acid	GPR150
palmitic acid	RHO
palmitic acid	RRM2
palmitic acid	RRM2B
palmitic acid	RPS6KB1
palmitic acid	RGR
palmitic acid	ANXA1
palmitic acid	OLAH
palmitic acid	SCARB1
palmitic acid	SEC14L2
palmitic acid	SEC14L2
palmitic acid	SEC14L2
palmitic acid	DBI
palmitic acid	STK40
palmitic acid	CDC42BPB
palmitic acid	MTOR

palmitic acid	NIM1K
palmitic acid	PIM1
palmitic acid	PIM2
palmitic acid	PIM3
palmitic acid	CD1D
palmitic acid	STK11
palmitic acid	ERN1
palmitic acid	ERN2
palmitic acid	PPP3CA
palmitic acid	PPP2R4
palmitic acid	PPP3CA
palmitic acid	ALB
palmitic acid	SAA1
palmitic acid	SAA2
palmitic acid	OPN1SW
palmitic acid	APLNR
palmitic acid	APOA1
palmitic acid	APOA2
palmitic acid	APOA4
palmitic acid	APOA5
palmitic acid	APOB
palmitic acid	APOC3
palmitic acid	APOE
palmitic acid	SLC2A2
palmitic acid	SLC22A8
palmitic acid	SLC22A11
palmitic acid	SLC22A5
palmitic acid	SLC22A6
palmitic acid	SLC22A8
palmitic acid	APOM
palmitic acid	BAX
palmitic acid	SSTR1
palmitic acid	SSTR2
palmitic acid	SSTR4
palmitic acid	SSTR5
palmitic acid	BCL2
palmitic acid	DEGS1
palmitic acid	FDFT1
palmitic acid	SREBF1
palmitic acid	SREBF2
palmitic acid	N/A
palmitic acid	MFN1
palmitic acid	NCOR2
palmitic acid	GPR161
palmitic acid	ARF6
palmitic acid	N/A
palmitic acid	CSMD1
palmitic acid	N/A
palmitic acid	CLEC2L
palmitic acid	MTRNR2L12
palmitic acid	TMIGD3
palmitic acid	IRAK4
palmitic acid	FADS1
palmitic acid	PLA2G2C
palmitic acid	TACR2
palmitic acid	TACR1
palmitic acid	SOD1
palmitic acid	SMARCD3
palmitic acid	KLRG2

palmitic acid	MATK
palmitic acid	SDC1
palmitic acid	SDC2
palmitic acid	SDC3
palmitic acid	SDC4
palmitic acid	TAC3
palmitic acid	CLEC5A
palmitic acid	GPR37L1
palmitic acid	CD1E
palmitic acid	TSSK1B
palmitic acid	TSSK2
palmitic acid	TSSK3
palmitic acid	TSSK4
palmitic acid	TXNRD1
palmitic acid	THBS1
palmitic acid	THBS2
palmitic acid	THBS3
palmitic acid	THBS4
palmitic acid	TBXA2R
palmitic acid	TPO
palmitic acid	TRHR
palmitic acid	TIAM2
palmitic acid	TLR2
palmitic acid	TLR4
palmitic acid	TAAR1
palmitic acid	TAAR2
palmitic acid	TAAR5
palmitic acid	TAAR6
palmitic acid	TAAR8
palmitic acid	TRAPPC3
palmitic acid	TRAPPC1
palmitic acid	TRAPPC2
palmitic acid	TRAPPC3
palmitic acid	TRAPPC3L
palmitic acid	TRAPPC4
palmitic acid	TRAPPC5
palmitic acid	TRAPPC6A
palmitic acid	TRAPPC6B
palmitic acid	ASGR1
palmitic acid	SP1
palmitic acid	TRIB1
palmitic acid	TRIB2
palmitic acid	TRIB3
palmitic acid	TGS1
palmitic acid	TYW1
palmitic acid	GLTP
palmitic acid	HNF4A
palmitic acid	TNF
palmitic acid	TNFRSF10B
palmitic acid	TNFRSF21
palmitic acid	AGTR1
palmitic acid	AGTR2
palmitic acid	ABL1
palmitic acid	ABL1
palmitic acid	ABL2
palmitic acid	BLK
palmitic acid	BTK
palmitic acid	CSK
palmitic acid	FGR

palmitic acid	FYN
palmitic acid	ITK
palmitic acid	JAK1
palmitic acid	JAK2
palmitic acid	JAK3
palmitic acid	LCK
palmitic acid	LYN
palmitic acid	MERTK
palmitic acid	TIE1
palmitic acid	TYRO3
palmitic acid	AXL
palmitic acid	SRMS
palmitic acid	STYK1
palmitic acid	SYK
palmitic acid	TEC
palmitic acid	ROR1
palmitic acid	TXK
palmitic acid	YES1
palmitic acid	ZAP70
palmitic acid	PTPN1
palmitic acid	ABCA1
palmitic acid	MLXIP
palmitic acid	UGT1A9
palmitic acid	ABCB1
palmitic acid	ABCE1
palmitic acid	ABCG1
palmitic acid	HELZ2
palmitic acid	MYO7A
palmitic acid	GPR17
palmitic acid	ABCB1
palmitic acid	ABCB1
palmitic acid	UTS2
palmitic acid	UTS2R
palmitic acid	UTS2B
palmitic acid	FLT1
palmitic acid	KDR
palmitic acid	FLT4
palmitic acid	ACLY
palmitic acid	LTB4R2
palmitic acid	AVPR1A
palmitic acid	AVPR1B
palmitic acid	AVPR2
palmitic acid	AVP
palmitic acid	SLC27A2
palmitic acid	RRH
palmitic acid	XBP1
palmitic acid	CR1L
palmitic acid	CLEC12A
palmitic acid	OPN5
palmitic acid	BDKRB1
palmitic acid	HSPG2
palmitic acid	CD72
palmitic acid	ABL1
palmitic acid	ADRB1
palmitic acid	HMGCR
palmitic acid	ADRB2
palmitic acid	APOH
palmitic acid	B2M
palmitic acid	ADRB3

palmitic acid	BAAT
palmitic acid	SULT2A1
palmitic acid	BRS3
palmitic acid	PRG2
palmitic acid	BMP4
palmitic acid	C4BPA
palmitic acid	C4BPB
palmitic acid	ABL1
palmitic acid	PPP3R1
palmitic acid	PLA2G5
palmitic acid	PRKACA
palmitic acid	PKIA
palmitic acid	PKIA
palmitic acid	CNR1
palmitic acid	CNR2
palmitic acid	MLXIPL
palmitic acid	CES4A
palmitic acid	CES5A
palmitic acid	N/A
palmitic acid	CRAT
palmitic acid	CPT1C
palmitic acid	CPT1A
palmitic acid	CPT1B
palmitic acid	CPT2
palmitic acid	COMP
palmitic acid	CASP6
palmitic acid	CTNNB1
palmitic acid	EPHA6
palmitic acid	CCR1
palmitic acid	CCR2
palmitic acid	CCR3
palmitic acid	CCR4
palmitic acid	CCR5
palmitic acid	CCR9
palmitic acid	CCL2
palmitic acid	CD209
palmitic acid	TP53
palmitic acid	UGCG
palmitic acid	ALAS1
palmitic acid	MAP3K20
palmitic acid	XCR1
palmitic acid	CCK
palmitic acid	CCKAR
palmitic acid	CYP7A1
palmitic acid	CHAT
palmitic acid	SLC44A1
palmitic acid	PCYT1A
palmitic acid	CHODL
palmitic acid	CHD9
palmitic acid	HTR1A
palmitic acid	HTR1B
palmitic acid	HTR1E
palmitic acid	HTR1F
palmitic acid	HTR2A
palmitic acid	HTR2B
palmitic acid	HTR2C
palmitic acid	HTR4
palmitic acid	HTR5A
palmitic acid	HTR6

palmitic acid	HTR7
palmitic acid	CLPS
palmitic acid	COL1A1
palmitic acid	CD55
palmitic acid	CTGF
palmitic acid	COG2
palmitic acid	CREBBP
palmitic acid	CLEC1A
palmitic acid	CLEC1B
palmitic acid	CLEC11A
palmitic acid	CLEC12B
palmitic acid	CLEC18A
palmitic acid	CLEC18B
palmitic acid	CLEC18C
palmitic acid	CLEC2A
palmitic acid	CLEC2B
palmitic acid	CLEC2D
palmitic acid	CLEC3A
palmitic acid	CLEC4A
palmitic acid	CLEC4E
palmitic acid	CLEC4G
palmitic acid	CD207
palmitic acid	CLEC4M
palmitic acid	GPR142
palmitic acid	CLEC6A
palmitic acid	CLEC7A
palmitic acid	CLEC9A
palmitic acid	CSMD2
palmitic acid	CSMD3
palmitic acid	CXCR4
palmitic acid	CXCR6
palmitic acid	CXCR7
palmitic acid	ATF4
palmitic acid	ATF6
palmitic acid	ATF6B
palmitic acid	CREB1
palmitic acid	CYSLTR1
palmitic acid	CYSLTR2
palmitic acid	CYCS
palmitic acid	CYP1A1
palmitic acid	CYP2B6
palmitic acid	CYP2C8
palmitic acid	CYP4A11
palmitic acid	XCL2
palmitic acid	BMX
palmitic acid	ACOT7
palmitic acid	DRD5
palmitic acid	DRD2
palmitic acid	DRD3
palmitic acid	DRD4
palmitic acid	OPRD1
palmitic acid	GNPAT
palmitic acid	ABL1
palmitic acid	DSTYK
palmitic acid	TESK1
palmitic acid	DNM1
palmitic acid	DNM1L
palmitic acid	DNM2
palmitic acid	OPA1

palmitic acid	CD69
palmitic acid	EGR1
palmitic acid	EDNRB
palmitic acid	EDN1
palmitic acid	EDNRA
palmitic acid	EDN2
palmitic acid	EDN3
palmitic acid	ACACB
palmitic acid	ECI2
palmitic acid	EPX
palmitic acid	EPHA1
palmitic acid	EPHA10
palmitic acid	EPHA2
palmitic acid	EPHA3
palmitic acid	EPHA4
palmitic acid	EPHA5
palmitic acid	EPHA7
palmitic acid	EPHA8
palmitic acid	EPHB1
palmitic acid	EPHB2
palmitic acid	EPHB3
palmitic acid	EPHB4
palmitic acid	EPHB6
palmitic acid	GPX5
palmitic acid	FABP4
palmitic acid	SELE
palmitic acid	EIF2AK3
palmitic acid	SLC1A3
palmitic acid	SLC1A2
palmitic acid	SLC1A1
palmitic acid	OC90
palmitic acid	SLC1A6
palmitic acid	SLC1A7
palmitic acid	FABP3
palmitic acid	FABP5
palmitic acid	FASN
palmitic acid	FABP5
palmitic acid	FABP4
palmitic acid	FABP5
palmitic acid	FABP3
palmitic acid	FABP2
palmitic acid	FABP1
palmitic acid	REG3G
palmitic acid	GPR173
palmitic acid	TBL1X
palmitic acid	TBL1XR1
palmitic acid	FGFR1
palmitic acid	FGFR2
palmitic acid	FGFR3
palmitic acid	FGFR4
palmitic acid	FGFRL1
palmitic acid	FKBP1A
palmitic acid	NPVF
palmitic acid	FOXO3
palmitic acid	FHL2
palmitic acid	FFAR1
palmitic acid	FFAR2
palmitic acid	FFAR3
palmitic acid	FURIN

palmitic acid	FURIN
palmitic acid	G0S2
palmitic acid	GALR1
palmitic acid	GALR2
palmitic acid	GALR3
palmitic acid	GM2A
palmitic acid	GIP
palmitic acid	GIPR
palmitic acid	GAST
palmitic acid	CCKBR
palmitic acid	GRP
palmitic acid	GRPR
palmitic acid	GLIPR1
palmitic acid	HRK
palmitic acid	GCG
palmitic acid	GCGR
palmitic acid	GLP1R
palmitic acid	GLP2R
palmitic acid	GCK
palmitic acid	GPR119
palmitic acid	GPX1
palmitic acid	GPX2
palmitic acid	GPX3
palmitic acid	GPX6
palmitic acid	GPX7
palmitic acid	PAEP
palmitic acid	SCD
palmitic acid	GSK3A
palmitic acid	GSK3B
palmitic acid	GLTP
palmitic acid	GLTP
palmitic acid	GLTP
palmitic acid	GPC1
palmitic acid	GPC2
palmitic acid	GPC3
palmitic acid	GPC4
palmitic acid	GPC5
palmitic acid	GPC6
palmitic acid	GNRHR
palmitic acid	GPR12
palmitic acid	GPR26
palmitic acid	GPR3
palmitic acid	GPR39
palmitic acid	GPR4
palmitic acid	GPR6
palmitic acid	GPR78
palmitic acid	GPR84
palmitic acid	GPRC6A
palmitic acid	GRHL1
palmitic acid	PLA2G10
palmitic acid	PLA2G2D
palmitic acid	PLA2G2E
palmitic acid	PLA2G2F
palmitic acid	ACBD7
palmitic acid	GHSR
palmitic acid	GNG2
palmitic acid	GNAQ
palmitic acid	GNA11
palmitic acid	GNA14

palmitic acid	GNA15
palmitic acid	ACOT1
palmitic acid	GUCA1A
palmitic acid	ACOT2
palmitic acid	ACOT4
palmitic acid	ACOT8
palmitic acid	LYPLA1
palmitic acid	HNF4A
palmitic acid	MET
palmitic acid	HNF4A
palmitic acid	HNF4G
palmitic acid	HAMP
palmitic acid	HK2
palmitic acid	HK3
palmitic acid	HRH1
palmitic acid	HRH2
palmitic acid	HRH3
palmitic acid	HRH4
palmitic acid	HAL
palmitic acid	HDAC3
palmitic acid	RNASEL
palmitic acid	CARM1
palmitic acid	ADORA1
palmitic acid	HNF4A
palmitic acid	HNF4G
palmitic acid	ADORA2A
palmitic acid	ADORA2B
palmitic acid	PSMC3IP
palmitic acid	HUNK
palmitic acid	HMGCS1
palmitic acid	HMGCS2
palmitic acid	PTK7
palmitic acid	INS
palmitic acid	INSR
palmitic acid	IRS1
palmitic acid	INSRR
palmitic acid	IGF1R
palmitic acid	IGF1
palmitic acid	IGF2
palmitic acid	ITGAX
palmitic acid	MX1
palmitic acid	MX2
palmitic acid	EIF2AK2
palmitic acid	IL1B
palmitic acid	IRAK1
palmitic acid	IRAK3
palmitic acid	IRAK2
palmitic acid	IL10
palmitic acid	ADIPOQ
palmitic acid	GPR135
palmitic acid	IL6
palmitic acid	CXCL8
palmitic acid	OPRK1
palmitic acid	ARF1
palmitic acid	ARF1
palmitic acid	ARF6
palmitic acid	KLRB1
palmitic acid	KLRG1
palmitic acid	KNG1

palmitic acid	KISS1R
palmitic acid	LPO
palmitic acid	LTB4R
palmitic acid	LTC4S
palmitic acid	MC2R
palmitic acid	LIMK1
palmitic acid	LIMK2
palmitic acid	REG1A
palmitic acid	REG1B
palmitic acid	CES1
palmitic acid	SLC27A1
palmitic acid	ACSL1
palmitic acid	ACSL3
palmitic acid	ACSL4
palmitic acid	ACSL5
palmitic acid	ACSL6
palmitic acid	ACSBG1
palmitic acid	OPN1LW
palmitic acid	FCER2
palmitic acid	LY96
palmitic acid	LSP1
palmitic acid	PBK
palmitic acid	XCL1
palmitic acid	KLRF2
palmitic acid	LPAR1
palmitic acid	LPAR2
palmitic acid	LPAR3
palmitic acid	LPAR5
palmitic acid	LPAR6
palmitic acid	PPT2
palmitic acid	SCARB2
palmitic acid	AGRN
palmitic acid	CSF1R
palmitic acid	CLECL1
palmitic acid	KIT
palmitic acid	MELK
palmitic acid	LY96
palmitic acid	MED1
palmitic acid	ACADM
palmitic acid	OPN1MW2 {ECO:0000312|HGNC:HGNC:26952}
palmitic acid	MCHR1
palmitic acid	MCHR2
palmitic acid	MC3R
palmitic acid	MC4R
palmitic acid	MC5R
palmitic acid	MC1R
palmitic acid	CLEC19A
palmitic acid	OPN4
palmitic acid	GRM1
palmitic acid	GRM5
palmitic acid	MT1E
palmitic acid	MT1F
palmitic acid	MT1H
palmitic acid	MT1X
palmitic acid	MT2A
palmitic acid	ALB
palmitic acid	KISS1
palmitic acid	MTRR
palmitic acid	CASR

palmitic acid	RAB19
palmitic acid	UCP2
palmitic acid	MFN2
palmitic acid	MAPK1
palmitic acid	MAPK11
palmitic acid	MAPK12
palmitic acid	MAPK13
palmitic acid	MAPK14
palmitic acid	MAPK15
palmitic acid	MAPK3
palmitic acid	MAPK4
palmitic acid	MAPK6
palmitic acid	MAPK7
palmitic acid	MAPK8
palmitic acid	MAPK9
palmitic acid	MAP3K10
palmitic acid	MAP3K11
palmitic acid	MAP3K7
palmitic acid	MAP3K9
palmitic acid	MLKL
palmitic acid	MLNR
palmitic acid	ABCB1
palmitic acid	ABCB4
palmitic acid	CHRM1
palmitic acid	CHRM3
palmitic acid	CHRM5
palmitic acid	MUSK
palmitic acid	OPRM1
palmitic acid	PMP2
palmitic acid	MPO
palmitic acid	AGPS
palmitic acid	SIRT1
palmitic acid	ME1
palmitic acid	NOX4
palmitic acid	POR
palmitic acid	NDOR1
palmitic acid	KLRD1
palmitic acid	NMB
palmitic acid	NMBR
palmitic acid	TACR3
palmitic acid	NMS
palmitic acid	NMU
palmitic acid	NMUR1
palmitic acid	NMUR2
palmitic acid	NPAS2
palmitic acid	NPFFR1
palmitic acid	NPFFR2
palmitic acid	ADRA1A
palmitic acid	NPS
palmitic acid	NPSR1
palmitic acid	NPY1R
palmitic acid	NPY2R
palmitic acid	PPYR1
palmitic acid	NPY5R
palmitic acid	NPBWR1
palmitic acid	NPBWR2
palmitic acid	NTSR1
palmitic acid	NTSR2
palmitic acid	NTS

palmitic acid	SLC1A4
palmitic acid	SLC1A5
palmitic acid	ASAH2
palmitic acid	FPR2
palmitic acid	ADRA1B
palmitic acid	ADRA1D
palmitic acid	NOS1
palmitic acid	NOS3
palmitic acid	NOS2
palmitic acid	KLRK1
palmitic acid	OPRL1
palmitic acid	TYK2
palmitic acid	ADRA2A
palmitic acid	ADRA2C
palmitic acid	N/A
palmitic acid	NCOA1
palmitic acid	NCOA2
palmitic acid	NCOA6
palmitic acid	NCOR1
palmitic acid	NR4A1
palmitic acid	NRF1
palmitic acid	NFYA
palmitic acid	NFYB
palmitic acid	NFYC
palmitic acid	TRAPPC2B
palmitic acid	RABL6
palmitic acid	O3FAR1
palmitic acid	OPN3
palmitic acid	QRFP
palmitic acid	HCRT
palmitic acid	HCRTR1
palmitic acid	HCRTR2
palmitic acid	GPR68
palmitic acid	OLR1
palmitic acid	OXTR
palmitic acid	OXT
palmitic acid	P2RY1
palmitic acid	P2RY2
palmitic acid	P2RY6
palmitic acid	SIN3A
palmitic acid	PPT1
palmitic acid	PDX1
palmitic acid	PNLIP
palmitic acid	LALBA
palmitic acid	PASK
palmitic acid	FKBP11
palmitic acid	FKBP1A
palmitic acid	FKBP1A
palmitic acid	PLIN2
palmitic acid	CASK
palmitic acid	PXDN
palmitic acid	PXDNL
palmitic acid	PEX11A
palmitic acid	PPARA
palmitic acid	PPARG
palmitic acid	PPARGC1A
palmitic acid	PPARGC1B
palmitic acid	ABCB1
palmitic acid	PTEN

palmitic acid	INPPL1
palmitic acid	PDE1C
palmitic acid	PLA2G2C
palmitic acid	PLA2G1B
palmitic acid	PLA2G2A
palmitic acid	PLB1
palmitic acid	GPX4
palmitic acid	SERPINE1
palmitic acid	CD36
palmitic acid	PTAFR
palmitic acid	PDGFRA
palmitic acid	PDGFRB
palmitic acid	PDGFRL
palmitic acid	PVR
palmitic acid	GM2A
